# Tissue-Engineered Grafts from Human Decellularized Extracellular Matrices: A Systematic Review and Future Perspectives

**DOI:** 10.3390/ijms19124117

**Published:** 2018-12-18

**Authors:** Andrea Porzionato, Elena Stocco, Silvia Barbon, Francesca Grandi, Veronica Macchi, Raffaele De Caro

**Affiliations:** 1 Department of Neuroscience, Section of Human Anatomy, University of Padova, Via A. Gabelli 65, 35121 Padova, Italy; andrea.porzionato@unipd.it (A.P.); silvia.barbon@yahoo.it (S.B.); veronica.macchi@unipd.it (V.M.); raffaele.decaro@unipd.it (R.D.C.); 2 L.i.f.e.L.a.b. Program, Consorzio per la Ricerca Sanitaria (CORIS), Veneto Region, Via N. Giustiniani 2, 35128 Padova, Italy; 3 Complex Operative Unit—Pediatric Surgery, Hospital of Bolzano, Via L. Bӧhler 5, 39100 Bolzano, Italy; frsncesca.grandi7825@gmail.com

**Keywords:** decellularization, human, cell colonization, regenerative medicine, body donation, transplantation, mesenchymal stem cells, extracellular matrix, scaffolds, biomechanics

## Abstract

Tissue engineering and regenerative medicine involve many different artificial and biologic materials, frequently integrated in composite scaffolds, which can be repopulated with various cell types. One of the most promising scaffolds is decellularized allogeneic extracellular matrix (ECM) then recellularized by autologous or stem cells, in order to develop fully personalized clinical approaches. Decellularization protocols have to efficiently remove immunogenic cellular materials, maintaining the nonimmunogenic ECM, which is endowed with specific inductive/differentiating actions due to its architecture and bioactive factors. In the present paper, we review the available literature about the development of grafts from decellularized human tissues/organs. Human tissues may be obtained not only from surgery but also from cadavers, suggesting possible development of Human Tissue BioBanks from body donation programs. Many human tissues/organs have been decellularized for tissue engineering purposes, such as cartilage, bone, skeletal muscle, tendons, adipose tissue, heart, vessels, lung, dental pulp, intestine, liver, pancreas, kidney, gonads, uterus, childbirth products, cornea, and peripheral nerves. In vitro recellularizations have been reported with various cell types and procedures (seeding, injection, and perfusion). Conversely, studies about in vivo behaviour are poorly represented. Actually, the future challenge will be the development of human grafts to be implanted fully restored in all their structural/functional aspects.

## 1. Introduction

In case of tissue injury or disease, tissue autografts are often considered the gold standard but inherent limitations including donor site morbidity, low availability, and unacceptable failure rates highlight the need for alternative strategies [2,3].

To date, engineering human tissues is an interdisciplinary and a very attractive field of research [4], but fully reproducing the properties of the extracellular matrix (ECM) is a great challenge [5,6]. Moreover, despite technological progress and advances in polymer science, the vast majority of artificial scaffolds do not satisfy the demand for a bioactive support, endowed with cell-instructive and cell-responsive properties [5]. 

The best scaffold for tissue engineering would be the decellularized ECM of the target tissue. In fact, decellularization allows researchers to obtain cell-free, natural ECMs characterized by an adequate 3D organization and proper molecular composition [7]. However, matrices may be beneficial also if not belonging to the same anatomical region, as demonstrated, for instance, by Kang and Colleagues [8], who considered decellularized nasal cartilage flakes for vocal fold augmentation. 

To date, both xenogeneic and allogeneic materials have been used in different hosts with different long-term results, but availability of donor tissues of consistent quality is often limited. Xenogeneic tissues from animals may carry residual immunogenicity and may be contaminated with biological agents. Human allogeneic tissues would be the ideal material to obtain ECM suitable for recellularization and implantation [9]. Human tissues/organs may be obtained from surgery or cadavers. In our experience, banking human tissues through the promotion of Body Donation Programs could help in the development of innovative strategies to recover injured tissues. The Body Donation Program of the University of Padua collects donated cadavers and body parts surgically removed [10,11]. Although this anatomical material is mainly used for didactic purposes, cadavers and body parts of body donation programs may also represent a valuable source of human organs and tissues for research as a human BioBank [12]. In collaboration with other clinical and surgical groups, we have developed a series of biologic scaffolds derived from decellularized human tissues from our Body Donation Program [13,14,15]. In our opinion, the role of Body Donation Programs should be implemented as Human Tissue BioBanks, which should be valuable sources of human tissues for graft development.

This review article outlines the importance of a readily available source of human tissues to develop ECMs for tissue engineering approaches. In particular, it considers main human-derived tissues that have been decellularized for tissue engineering purposes, detailing the different decellularization methods adopted, and if recellularization and in vivo implantation occurred. A suitable strategy for processing tissues should exclude the risk of infection and disease transmission, it should guarantee exhaustive decellularization for elimination of antigenicity and avoidance of immunoreactions and it should not affect the biomechanical integrity of the matrix, recellularization capability, or inductive properties. 

As acellular skin is already extensively used in clinical practice, as well as being available as a commercial product, it has not been taken into consideration.

## 2. Methods

This systematic review was performed in accordance with the statements and guidelines of the Preferred Reporting Items of Systematic Reviews and Meta-analyses (PRISMA) [16]. 

In particular, the PubMed database of the US National Library of Medicine and Scopus database were queried for the same search strings “human decellularization” OR “human acellular matrix tissue engineering”. The latest search was conducted on November 2018; duplicate studies were discarded screening only relevant articles according to title and abstracts. No time or language limits were adopted.

## 3. Results

After database searching, the total number of records was 2928, divided as described below for PubMed and SCOPUS, respectively. For the search string (“human decellularization”), the identified records were (n = 733) and (n = 1017); for the search string (“human acellular matrix tissue engineering”), the identified records were (n = 482) and (n = 696).

**Figure 1:**
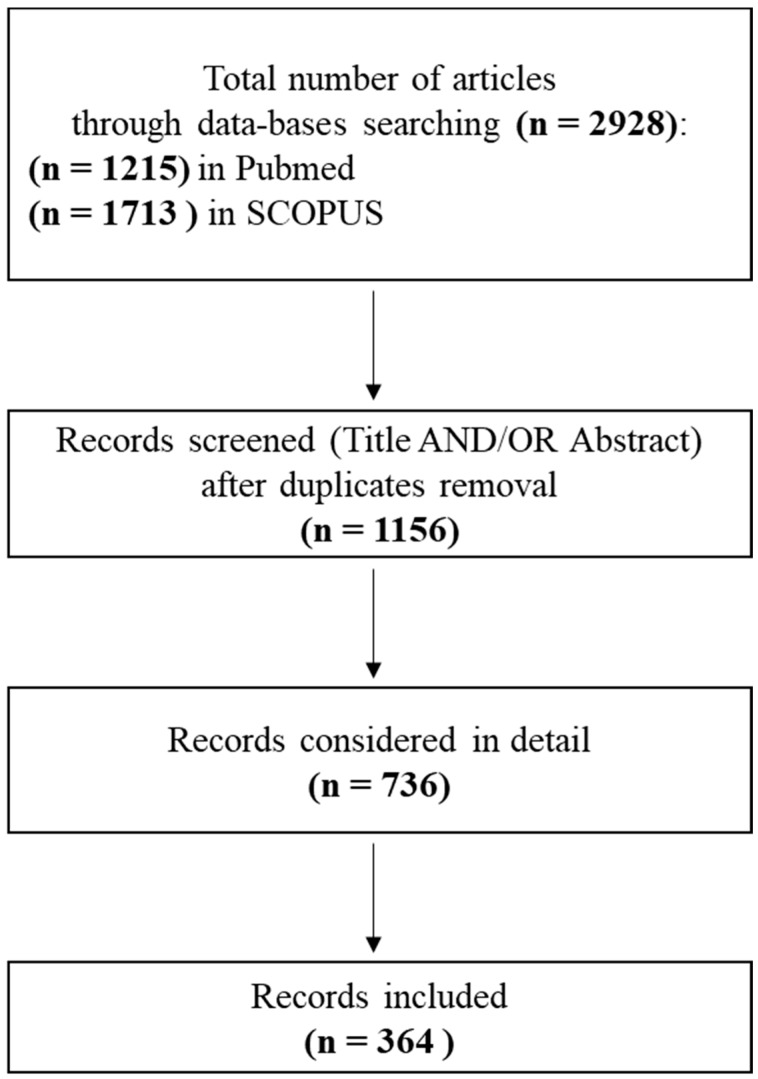
Flow diagram showing the literature search criteria adopted.

After screening titles and abstracts, and removing the duplicates as well as nonrelevant records, the decellularized tissues and organs of human origin referred to in the literature were selected and considered for this systematic review (Figure 1). 

## 4. Cartilage 

Cartilage lesions are usually caused by disease, trauma, and aging-related degenerations; however, congenital alterations may also occur [17,18,19,20,21,22,23]. Although decellularization is a good strategy to obtain an adequate and cell-instructive scaffold, in case of cartilages their compactness may represent an obstacle both to decellularization and repopulation [24]. However, various human cartilaginous structures have already been decellularized, including articular [25], meniscal [20,26], laryngeal [27], tracheal [28,29,30,31], and nasoseptal cartilages [8,32]. 

### 4.1. Hyaline Cartilage

**Figure 2:**
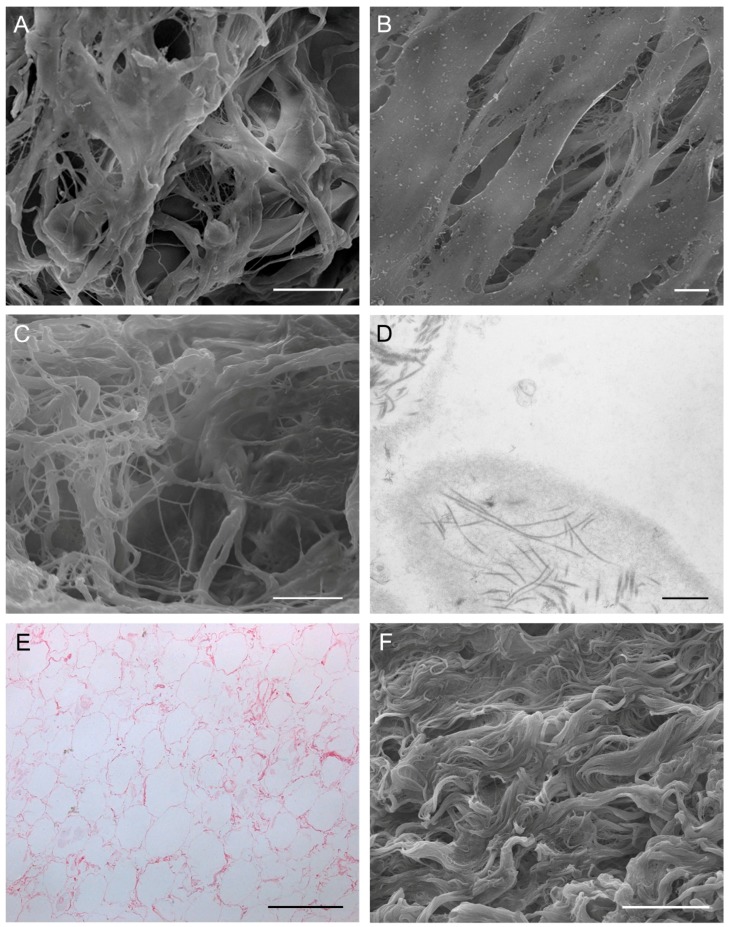
Decellularized human extracellular matrices. Ultrastuctural appearance: scanning electron microscope image of an articular cartilage scaffold before (**A**) and 14 days after primary human chondrocytes seeding (**B**). Human cartilage was decellularized, homogenated, and lyophilized according to the experience of our group. Scanning (**C**) and transmission (**D**) electron microscope micrographs of decellularized skeletal muscle. Hematoxylin/eosin staining (**E**) and scanning electron microscope ultrastructural analysis (**F**) of decellularized omentum. Scale bars: (**A**,**F**), 50 µm; (**B**,**C**), 10 µm; (**D**), 1 µm; (**E**), 100 µm.

To date, a wide variety of decellularization techniques have been investigated, although there is no consensus about the optimal procedure [21]. In addition to physical and osmotic treatments, chemicals (i.e., sodium dodecyl sulfate (SDS), sodium lauryl sulfate, ethylenedimine tetraacetic acid (EDTA), Triton X-100, and Tris-HCl) and enzymes (DNases and RNases) are frequently used and combined in multistep procedures but, despite numerous cycles, some residual DNA may remain in the treated tissue [23,33]. To optimize decellularization, many authors also mechanically fragmented or pulverized the cartilage; increasing surface area promotes permeation of solutions, reduces exposure times to decellularizing agents, and preserves the microstructure, polysaccharide-like glycosaminoglycans (GAGs), and structural proteins including collagen II [33,34]. Interestingly, freezing/thawing (FT) cycles also improve the decellularization process allowing for more pores in the tissue after ice crystal formation [35]. After cartilage deconstruction, rebuilding the scaffold is necessary [35]. For istance, Stocco et al. [36] freeze-dried and lyophilized the ECM suspension which was previously homogenized in a 10% acetic acid solution (2.5 M) (Figure 2A,B); Yang et al. [24] did the same and then cross-linked it. Schneider et al. [25] compared 24 different protocols for decellularization of human articular cartilage, identifying the best one in FT cycles followed by further steps in hypotonic buffer (Tris-base, pH8), 0.1 M HCl, 1 g/L pepsin, and peracetic acid. 

Considering the larynx [27], it was decellularized according to a method that was previously set up for the trachea. In fact, as regards tracheal cartilage, Macchiarini et al. [28], Gonfiotti et al. [37], Baiguera et al. [38], and Elliott et al. [30] experienced a detergent enzymatic approach by means of 4% sodium deoxycholate, dH_2_O, and 2000 KU (Kunitz Units) DNase-I in 1 M NaCl followed by further washes in dH_2_O. A slight change to this protocol was recently reported; in fact Tint et al. [39] worked with tracheas which were previously freezed in liquid nitrogen and rinsed in a solution of 2 mM CaCl_2_ and 1.3 mM MgSO_4_ after DNase treatment. However, in all the reported experiences, a complete decelluarization of human trachea required several cycles for a period of three to eight weeks. Hence, Butler and Collegues [29], aiming to fasten decellularization, described an accelerated vacuum-assisted method. After allocating the tissue in a Ricordi chamber, vacuum (1 Torr) was generated allowing for a better penetration of the detergent enzymatic solutions in the dense trachea cartilage. The method guaranteed for a reduction in decellularization time (nine days) with respect to common protocol reported above, without vacuum.

The necessity for an ideal scaffold for dorsal augmentation in rhinoplasty and the absence of a single ideal material to this purpose [40] suggests that decellularized human cadaver-derived nasal cartilage may represent an interesting perspective. To date, nasal reconstruction in patients who are missing a significant amount of structural nasal support remains a difficult challenge. Historically, the standard donor source for large quantities of native cartilage has been costal cartilage [41] but some studies also proposed tissue engineering approaches from human cadaver nasal cartilages [42]. Schwarz et al. [32] developed a cost-effective and simple decellularization method based on osmotic treatment in distilled water (dH_2_O) and standard chemicals (sodium hydroxyde, ethanol, guanidine hydrochloride, sodium acetate, and hydrogen peroxide), without any enzymatic digestion. Later, Kang and Colleagues [8] also considered the decellularization of cadaver nasoseptal cartilage to obtain material for vocal fold augmentation; they used Tris/EDTA with protease inhibitor, 1% Triton X-100 or 2% SDS, and DNAse/RNase. 

Neither Schwarz et al. [32] nor Kang et al. [8] investigated possible recellularization approaches but recellularization was studies for other cartilage types. As it regards decellularized articular cartilage, cytocompatibility tests were performed with human adipose-derived stromal cells, which attached to the scaffold [25], and recellularizations were achieved using mesenchymal stem cells (MSCs) or primary chondrocytes [36,43,44]. 

Considering tracheal scaffold, human engineered tracheas implanted in patients were previously repopulated by epithelial cells from bronchi or inferior turbinate mucosa and bone marrow MSCs [28]. However, to minimize delays as well as the risk of malignancy [30,45], some Authors proposed to seed MSCs onto the scaffold endowed with autologous epithelium patches. For the tracheal repair, another suitable source of MSCs could be represented by adipose-derived MSCs, but so far there are only a few reports about their use in airways tissue engineering [46,47]. Actually, whether the seeding of stems cells is necessary for successful tracheal scaffold transplantation is still debated. Hung et al. [48] sustain that the main challenge in achieving transplantation success is related to the maintenance of scaffold integrity, while Go et al. [49] stress the importance of recellularization. Wood et al. [50] showed that the survival of the animals implanted with the stem cell-seeded trachea scaffolds was not longer than the matrix-only group.

Regarding clinical efficacy studies, case reports exist about implantation of bioengineered human tracheas in patients (Table 1); the recipients were a 30 years old woman [28,37] and a 10 years old boy [30,31]. In both cases it was a compassionate use procedure which took advantage from a deceased donor trachea decellularized by a detergent enzymatic method and then repopulated with autologous cells/tissue patches. The treatment assured for patient survival and increase of life expectancy. 

### 4.2. Fibrocartilage (Menisci)

Some studies addressed decellularization and repopulation of human menisci. As it regards decellularization protocols, Sandmann et al. [26] used solutions containing various concentrations of SDS (1%, 2%, and 5%) for one or two weeks; complete cell removal, without compromising compressive properties, was achieved with 2% SDS after two weeks. Minehara et al. [51] used incubations in acetone, hydrogen peroxide, and osmotic solutions. Nordberg et al. [52] adopted 24 h enzymatic digestion in 0.05% trypsin EDTA and 48 h incubation in 2% Triton X-100 and 1.5% peracetic acid, apart from other steps in deionized water and neutralization medium. Homogenizing approaches have also been proposed [53].

Repopulation of decellularized menisci has been performed with rat chondrocytes [51], human adipose-derived stem cells [52], and human synovial fluid-derived mesenchymal stem cells [53]. Various authors have stressed the difficulties in the recellularization of menisci, due to the dense structure of the extracellular matrix with very few interstitial spaces [51,52]. Consistently, some authors proposed chemotactic cell seeding techniques (recombinant human bone morphogenetic protein-2) [51] or needle punching (1-mm spacing, 28G microneedle) [52] to facilitate cell invasion. Exogenous growth factor supplementation (TGF-β_3_ and IGF-1) was required to chondrogenic differentiation of synovial fluid-derived mesenchymal stem cells [53].

### 4.3. Elastic Cartilage (Auricular Cartilage)

Auricular elastic cartilage has also proved to be quite difficult to be decellularized due to its dense structure. Some studies addressed decellularization of cartilage fragments after removing of skin and perichondrium [54,55]. Utomo et al. [54] employed freeze–thaw cycles (dry and in hypotonic buffer), and following incubations in hypotonic buffer, in 0.1% SDS and 0.1% EDTA, in elastase and in nuclease (DNAse and RNAse) solutions. Rahman et al. [55], instead, recently compared three different protocols. Protocol A involved dry and wet freeze–thaw cycles followed by incubations in 4% SDS and 2% DNAse solutions. Protocols B and C involved additional incubations in 0.25% trypsin or 0.5 M EDTA, respectively, after protocol A. Protocol B proved the most effective in the decellularization process.

Differently from the above approaches, a recent work considered decellularization of whole ears, comprehensive of skin and vascular tree [56]. Decellularization was performed through arterial perfusion involving the following solutions, heparinized saline containing 10 µM adenosine, 1% SDS, 1% Triton X-100, 100% 2-propanol, and 50 UI/mL DNAse I.

In this study, good biocompatibility was observed after subcutaneous implantation of decellularized ear fragments in rats for up to 60 days [56]. Good in vitro recellularization was also achieved with seeding of rat adipose-derived stem cells and perfusion of human aortic endothelial cells, although repopulation of the cartilage remains a challenge. 

## 5. Bone

As a consequence of surgical procedures or chronic diseases, bone grafting may be necessary to reconstruct skeletal defects. Resorting to autologous bone is the “gold standard” but this approach is not free from issues, such as availability, donor site morbidity, infections, prolonged wound drainage, postoperative pain, and neurovascular injuries [34]. To overcome these limits, allografts (mainly donated femoral heads) are commonly used but the risk of immune response and pathogen transfer have prompted the research towards tissue substitutes like decellularized bone [80,81]. 

According to our knowledge, only two research groups considered the decellularization of human bone. Smith and Collaborators [82,83] developed a method based on wash–centrifuge phases (i.e., dH_2_O preheated to 60 °C under shaking, followed by centrifugation at room temperature) and sonication (sterilant solution at 60 °C and then in 70% *v*/*v* ethanol at 21 °C). The resulting material was free from the marrow elements, which may interfere with the graft osteointegration. Moreover, according to in vitro assays, it sustained viability and osteogenic activity of human bone marrow MSCs without need for osteogenic medium, suggesting the maintenance of functional ECM proteins and growth factors [82]. Then, decellularized human bones from donors of different ages were seeded in vitro with human bone marrow MSCs from young or old donors; it emerged that old donor bones were better in promoting osteogenic differentiation of MSCs than the young ones. While, regarding cells, MSCs from younger donors showed a more differentiated cell phenotype than the others [83]. Later, Sladkova et al. [84] proposed a protocol that required an incubation in 0.1% EDTA buffer followed by detergent and enzymatic solutions (0.1% EDTA in 10 mM Tris, 0.5% SDS in Tris, and 100 U/mL DNase/RNase in Tris buffer) to remove cellular material from cadaveric human bone. The scaffold was conditioned with osteogenic medium and seeded with human induced pluripotent stem cells-derived mesenchymal progenitor (iPSC-MP) prior to be transferred to perfusion bioreactor. After five weeks, the scaffold demonstrated its adequacy in supporting cell viability and osteogenic differentiation as well as bone specific matrix deposition.

## 6. Skeletal Muscle

Skeletal muscle losses due to traumatic injuries or infective or neoplastic pathologies represent a clinical problem which is usually overcome with transfers of autologous muscle tissue or muscle flaps. These procedures, however, are associated with donor site morbidity and are not always possible. On the other hand, xenografts and allografts are associated with the risk of immune response and worse integration. Thus, the development of engineered skeletal muscle grafts from homologous ECM and autologous cells has recently been proposed for replacing volumetric muscle losses (Table 2). Many works have been performed with animal models (reviewed, for instance, in Urciuolo and De Coppi [85]), but few authors have considered the decellularization of human skeletal muscles. In a previous study, we decellularized human skeletal muscle samples taken from amputated limbs (tibialis anterior) and cadavers (abdominal rectus muscle) [14]. Complete removal of skeletal muscle cells was achieved with a protocol involving 1 h incubation in 0.05% trypsin with 0.02% EDTA and 72 h incubation in 2% Triton X-100 and 0.8% ammonium hydroxide (NH_4_OH); partial persistence of myofibrils being instead found with 4% SDS and DNase I. 

In vitro recellularization was not performed, however assessment of in vivo cell colonization was performed in rabbits through the suturing of the muscle graft to close a surgical defect of the abdominal rectus muscle. The muscle graft prevented visceral herniation and did not show signs of rejection or systemic infection; fibroblast invasion and neovascularization were found at sacrifice, three weeks after surgery, together with aspects of initial proliferation/migration of muscle fibers/progenitor cells at the periphery of the implant (Figure 2C,D). 

Later, Wilson et al. [86] described the decellularization of human rectus femoris and supraspinatus muscles. The tissues were freezed-thawed, soaked in 1% SDS with 1% EDTA in Tris–HCl buffer and then incubated in 1 kU/ml DNase/RNase buffer. As highlighted by the authors, the age of the patient is probably a limit of the study; however, analysis of decellularized tissues showed that the properties of the materials depend on the type of muscle: the rectus femoris is less elastic and richer in collagen than the supraspinatus. In addition, the in vivo degradation rate of decellularized muscles was also considered by implanting them into the subcutaneous dorsal pocket of mice for 4, 8, and 16 weeks. The collected data demonstated that the scaffolds biodegrade at a rate that is consistent with the reported regeneration of the damaged muscle. This is a significant aspect to consider, as biological scaffolds has not to be intended as permanent implants; rather, as a temporary support subject to ECM turnover by resident cells.

According to the literature, the hemidiaphragm of a deceased donor was also considered for tissue engineering purposes. Davari et al., [87] evaluated the effectiveness of cryopreservation versus decellularization before implanting derived hemidiaphragm patches in a canine defect model. Briefly, cryopreservation consisted in freezing the grafts at −80 °C and preserving them for up to 2 months; while decellularization was performed according to a detergent–enzymatic protocol consisting of a dH_2_O soaking-phase followed by 4% sodium deoxycholate and 2000 kU of DNase-I in 1 mol/L NaCl. After six months, samples were explanted and analyzed. Both grafts guaranteed a similar gross healing process, even if a lower infiltration of inflammatory cells and granulomas on histology was assured by decellularized patches. 

## 7. Tendon

Efficient decellularization of human tendons has been reported by various authors with different protocols (Table 2) [88,89,90,91,92,93,94,95,96]. The tendons used are usually upper limb flexor tendons or Achilles tendons sampled from cadavers or body parts removed during surgery. 

Recellularizations of tendon grafts have been successfully performed with human dermal fibroblasts [88,89], adipose-derived mesenchymal stem cells [91,92,93], and bone marrow-derived mesenchymal stem cells [94]. Regarding methods of recellularization, preliminary injection of collagen solution has been reported to improve the penetration of injected adipose-derived mesenchymal stem cells [92]. Injections of cells with fetal bovine serum or hydrogel have also been reported to improve cell colonization of decellularized tendons [93]. Le et al. [94] evaluated the effect of suture seeding with bone marrow-derived stem cells in an ex vivo tendon repair model. Woon et al. [89] also reported that peracetic acid increases scaffold porosity and improves cell penetration and migration; while Whitlock and Colleagues [95] observed that it does not compromise tensile properties of the scaffold neither renders the scaffolds cytotoxic or provokes an inflammatory response in vitro.

Few studies have developed implant approaches to evaluate the surgical suitability of the tendon grafts. Suppression of immunogenicity of decellularized human tendons was confirmed, for instance, in immunocompetent rats subjected to implants in the dorsal subcutaneous tissue [90]. Reseeded and unseeded repaired tendons were also biomechanically compared after implantation in dorsal subcutaneous tissue in athymic rats for two or four weeks, demonstrating the absence of significant differences in ultimate load to failure [91]. According to our knowledge, only Whitlock et al. [95] evaluated the efficacy of decellularized Achilles tendon for anterior cruciate ligament reconstruction in a rabbit model of injury. 

Decellularization of composite flexor tendon–bone interface grafts have also been performed in order to evaluate the suitability of such scaffolds to repair complex extremity injuries investing tendon attachment to bone [97,98]. Decellularized grafts showed no significant difference with respect to untreated samples in terms of ultimate failure load and stiffness. Further studies, however, will have to consider the eventual effects of recellularization and its surgical suitability in large animal models and humans.

Apart from tendon or tendon–bone grafts, decellularization of human tendons has also been employed to develop ECM gels, which can be delivered percutaneously into tendon injuries or may be used as vehicle for cell delivery [96,99].

## 8. Adipose Tissues

In reconstructive surgery and regenerative medicine, tissue implants are frequently needed for postoperative, congenital or post-traumatic loss of adipose tissue, which may result in scar tissue formation, deformity, and loss of function. Thus, tissue-engineered adipose substitutes have been proposed through decellularization of adipose tissue and possibly recellularization with autologous cells (Table 3). 

Among structures with high content of adipose tissue, we proposed for the first time the decellularization of the human greater omentum collected from cadavers [13]; other authors were performing similar approaches with porcine material [100,101]. To the best of our knowledge, there are only other two studies which considered the human omentum as a source of adipose tissue acellular matrix [102,103]. Autologous greater omentum is used in various surgical procedures, because of its rich vascularity, high angiogenic activity, innate immune function, ability to adhere to local structures, and high production capability of growth factors. Decellularization of greater omentum has been obtained through a detergent-free protocol involving mechanical rupture, polar solvent extraction, and enzymatic digestion [13,102,103]. The procedure was mainly derived from previous studies on decellularization of adipose tissue [104] and demonstrated to give rise to a complex acellular scaffold made up of a three-dimensional network of ECM and decellularized vascular bed maintaining collagen and elastic fiber structure [13,100,101] (Figure 2E,F). Recellularization experiments have been carried out on decellularized porcine omentum grafts [100,101], in order to highlight the good adhesive/proliferative properties of the tissue. However, the recellularization of human scaffolds has been poorly investigated, only Baker and Collaborators [103] reported the 3D-culture of human preadipocytes on acellular omentum. The study aimed at defining the role of the visceral adipose tissue ECM in regulating adipocyte metabolism in diabetes. The ECM demonstrated to support the differentiation of mature adipocytes, restoring or impairing their glucose uptake and lipolysis capacity depending on whether it was isolated from healthy subjects or diabetes patients, respectively [103].

Most studies have considered the decellularization of human adipose tissue samples harvested from routine operations (i.e., liposuction, reduction mammoplasty or abdominoplasty procedures, reconstructive surgery, bariatric surgery, and elective cosmetic surgery) in different districts (abdomen, breast, and forearm) [89,90,104,105,106,107,108,109,110,111,112,113,114,115,116,117,118,119,120,121,122,123,124]. For human adipose tissue decellularization, the less common sampling sources were pericardial depot and thymic remnants collected during surgery [102], as well as specimens from human cadavers [125] (Table 3). 

The protocol for human adipose tissue decellularization was first standardized by Flynn [104], consisting of a combination of mechanical, chemical and enzymatic treatments which extract cells and lipids, preserving the structural ECM components. Several authors performed subcutaneous tissue decellularization according to this detergent-free method, obtaining complete removal of the cellular fraction and maintenance of ECM structure and composition [102,107,108,112,113,114,115,116,117,119,120,121,126]. Besides turning to the detergent–enzymatic method [89,106,118], alternative decellularization approaches combined the mechanical disruption of the tissue (i.e., through homogenization or freezing–thawing) and enzyme digestion (i.e., by DNase, RNase, pancreatin, or trypsin), with [105,109,111,124,127] or without [110,120,123] the use of detergents (i.e., SDS, sodium deoxycholate, and Triton X-100). Some authors reported simplified protocols which involve the use of a singular category of decellularization agent, such as mechanical disruption by freezing–thawing [126] or immersion into detergent solutions [121,125]. Finally, Thomas-Porch and coworkers [121] introduced the use of a urea-based solvent method; also adopted by Li and colleagues [122] with the addiction of pepsin digestion treatment.

Interestingly, many reviewed studies have focused on designing injectable adipose-derived scaffolds with predefined shape and volume, in order to offer a minimally-invasive strategy for soft tissue augmentation in the clinical practice. To this end, after decellularization procedure, human adipose tissue was furtherly processed into different formulations: (a) hydrogel scaffolds made of adipose ECM alone [106,124] or in combination with chitosan [113], chondroitin sulphate [113,115,117], fibroin [118], or poly(ethylene glycol) [122]; (b) injectable adipose ECM microparticles [110,111]; (c) microcarriers consisting of acellular adipose matrix alone [116] or mixed with alginate [107,108]; (d) bead foams [112,119]; and (e) injectable acellular adipose matrix powder [125].

For decellularized adipose scaffold repopulation, human adipose-derived stem cells represented the prevailing choice [104,106,107,108,109,110,111,112,113,115,116,122,123,124], due to their availability and capability of differentiation in various cell types, including endothelial and adipose cells [13]. The recellularization with adipose-derived stem cells of rat origin was also reported for following autologous implant purposes [114,118]. Cell populations were either encapsulated into adipose ECM-based hydrogels or seeded in static/dynamic conditions onto acellular adipose matrix slices/microparticles/microcarriers/bead foams. Besides being nontoxic for adipose-derived cultures, decellularized ECM demonstrated not only to support cell adhesion and proliferation, but also to provide an inductive microenvironment for adipogenesis.

Differently, Dunne, and collaborators [127] reported the seeding of human adipose tissue-derived ECM matrix scaffolds with breast cancer cells, with the aim to reproduce in vitro a three-dimensional cell culturing system for the investigation of breast cancer growth and drug treatments. Furthermore, chronic wound human fibroblasts seeding onto adipose-derived bead foams under stress conditions served to prove the influence of adipose ECM composition on the modulation of wound healing response by enhancing cell survival and angiogenesis [119].

Finally, in vivo experiments were accomplished by performing subcutaneous implantation/injection of seeded and unseeded adipose tissue-derived scaffolds into athymic [106,119,125], nude [110], immunocompetent [105,120,122,125] or GFP^+^ transgenic mice [121], as well as nude [106,111] or immunocompetent rats [108,109,112,113,114,118,124]. In vivo studies demonstrated high biocompatibility of seeded and unseeded decellularized adipose scaffolds, with no evidence of inflammation and rejection upon subcutaneous implantation [124]. When seeded with adipose-derived stem cells, adipose tissue grafts integrated with the host tissue, promoting angiogenesis and fat formation, as well as supporting cell infiltration and tissue remodeling [108,109,111,112,113,114,125]. However, non-repopulated scaffolds also proved to be capable of supporting the in-growth of host-derived adipocytes progenitors and vasculature [121].

## 9. Heart

Cardiovascular diseases (CVD) represent one of the leading causes of death worldwide, and myocardial infarction is responsible for most of CVD-related mortality [128]. Given the shortage of suitable donor organs and the difficulties associated to risks of rejection and lifelong immunosuppressive therapy, there is an urgent need to improve the current clinical strategies [129]. In order to block the progression and induce repair of infarcted myocardial tissue, researchers are addressing particular attention to novel therapeutic approaches offered by cardiovascular regenerative medicine. Cell therapy with a variety of stem populations (i.e., cardiac stem/progenitor cells, bone marrow- and adipose tissue-derived stem cells, induced pluripotent stem cells) has largely been investigated as a promising regenerative strategy [130]. However, some important limitations such as low rate of engraftment and poor survival of stem/progenitor cells after transplantation have precluded their clinical application [131]. To enhance stem cell regenerative potential, their combination with adequate scaffolds could represent an improved therapeutic approach. Recently, several biomaterials have been investigated as scaffolds for cardiovascular tissue repair, ranging from synthetic polymers [132,133] to natural matrices [134]. Among these, decellularized myocardium and pericardium are arousing increasing interest for cardiac tissue engineering applications.

### 9.1. Myocardium

In the heart, the ECM is responsible of several pathophysiologic responses, including fibrosis, inflammation, angiogenesis, cardiomyocyte contractile function and viability, and resident progenitor cell fate [135]. For this reason, the importance of using tissue-specific matrix for generation of biological cardiac scaffolds is increasingly being acknowledged. To this end, decellularization of myocardial tissue of rat or pig origin has been investigated in depth [136]. Over the last decade, significant efforts have been directed to obtain successful decellularization of human myocardium with high regeneration potential. Specifically, regenerative strategies are meant to replace necrotic cardiomyocytes and/or repair damaged extracellular matrix. This is achieved by applying cardiac myocardial patches to the epicardial surface or injecting them directly into the myocardium to improve wall stress [137]. The myocardial ECM patch may work as a cell delivery platform which promotes survival and engraftment of cellular elements and possibly guides their differentiation for the replacement of necrotic tissue [138].

Human heart decellularization has been applied to whole organs [137,139,140,141] or cardiac muscle fragments [142] harvested from cadavers and unusable for clinical transplantation, as well as to tissue sections procured during surgical procedures (i.e., implantation of a mechanical assist device, heart transplantation, and resection of a subaortic outflow tract obstruction) [135,138,143,144,145,146,147,148]. Left ventricular biopsies derived from myocardial infarction patients were also processed [138]. In the attempt to generate human cardiac ECM scaffolds as cell delivery platforms for cardiac repair, several decellularization methods have been tested (Table 4). Perfusion decellularization of whole human hearts via antegrade flow through the ascending aorta or coronary arteries was achieved by the use of both detergent (SDS) [137,139,140] and detergent–enzymatic (SDS, Triton X-100/DNase) solutions [141]. Myocardial sections were decellularized by employing the same ionic (SDS, deoxycholic acid, sodium deoxycholate) [135,137,138,139,140,141,142,143,144,145,146,147,148] and non-ionic detergents (Triton X-100) [138,148], with [135,141,142,143,144,145,146,147,148] or without [137,138,139,140,148] the addiction of enzyme treatment. Interestingly, other than direct endonuclease incubation, the intrinsic DNase activity of foetal bovine serum was experienced to eliminate residual DNA [135,138,143,144,148]. Overall, perfusion decellularization of whole heart assured the maintenance of 3D organ macro/microstructure and vascular tree, together with the successful removal of cell material [137,141]. Notably, the decellularization process did not affect the anisotropic behaviour of myocardium upon passive mechanical testing, indicating the preservation of the native myocardial ECM structure [137,141].

Similarly, decellularization protocols of heart slices allowed to obtain cell-free scaffolds preserved in both protein composition and tissue architecture [135,138,143,145,148]. Considering evaluation of mechanical properties, acellular myocardial sheets maintained the characteristics of a relaxed native tissue [145].

After decellularization treatment, some authors [135,143] also described the processing of cardiac ECM sheets into a microparticle powder by mechanical grinding, or, via pepsin digestion, into a self-assembling cardiac ECM hydrogel with preserved bioactivity. These ECM formulations hold augmented potential of in vitro and in vivo applications for cardiac regeneration purposes [135]. The cell-support characteristics of this cardiac ECM were tested through murine HL-1 cardiomyocyte cultures. In particular, ECM microparticles were suspended in the culture medium or embedded in gelatin to coat the culture plate surface. When grown on ECM microparticles in normoxia conditions, HL-1 cells showed enhanced metabolic activity and proliferation. During simulated ischemia (i.e., hypoxia and glucose/serum deprivation), the myocardial matrix microparticles exerted specific cytoprotective effects on cardiomyocytes [143]. A further step in the investigation of this myocardial ECM hydrogel consisted of manufacturing a novel composite scaffold by combining the gel itself with acellular amniotic membrane via a dry coating method [144]. The resulting patch proved to support the proliferation of human cardiac fibroblasts, epicardial progenitor cells and murine HL-1 cardiomyocytes, as well as to exert immunomodulatory effects on human immune cells derived from buffy coat (monocytes, macrophages, and peripheral blood mononuclear cells) [144].

An injectable form of the human myocardial matrix was fabricated also by Johnson and colleagues [146], with the aim to perform absolute protein content quantification of the decellularized human cardiac tissue by global proteomics analysis. The study highlighted significant patient-to-patient variability between the investigated subjects, providing important information for the development of allogeneic derived biomaterials and for increasing the understanding of human myocardial ECM composition [146]. Moreover, Wang and coworkers [147] evaluated the immune response towards myocardial ECM hydrogels of human origin after injection into the subcutaneous dorsal tissue of humanized mice. The in vivo study demonstrated the importance of this animal model in the investigation of biocompatibility and pro-remodeling qualities of biomaterials before clinical application.

To be of clinical value, the acellular myocardial matrix should exhibit biocompatible and nonimmunogenic features. In order to assess these ECM properties, successful repopulation of decellularized scaffolds may represent a critical step before preclinical translation. Based on that, Godier-Furnémont and coworkers [145] considered fabricating a composite scaffold where human mesenchymal progenitor cells were embedded into fibrin hydrogel loaded onto decellularized myocardium sheets. In the composite, acellular myocardium provides a structural and biomechanical support, whereas fibrin enables cell retention and local signalling.

Innovative composite myocardial-based constructs were prepared by Guhathakurta et al. [142], through the coating of acellular cardiac ECM with electrospun poly-(l)lactic acid/polycapronolactone/collagen nanofibers and subsequent injection of cord blood mononuclear cells. The engeneered myocardial patches exhibited contractile activity and specific troponin I and cardiac myosin expression during in vitro culture.

Repopulation of acellular myocardial scaffolds was successfully accomplished by seeding cord blood-derived mesenchymal stem cells [138], bone-marrow mesenchymal stem cells [137], human cardiac progenitor cells [137,148], human endothelial cells (HUVECs) [137], cardiomyocytes derived from induced pluripotent stem cells [138,140,141], and neonatal mouse cardiomyocytes [138]. Decellularized human heart was also cell-repopulated as a whole organ by perfusion of cardiomyocytes derived from induced pluripotent stem cells [141]. Remarkably, cardiomyocytes seeded on the myocardial matrix coalesced over time into nascent cardiac muscle fibres [137], ending to produce spontaneously contracting slices [141], whereas endothelial cells were found lining the vascular conduits [137]. Moreover, the differentiation of cardiac progenitor cells towards myocardium and smooth muscle cell lineage [148] was also achieved. In short, scaffold recellularization studies documented that the microenvironment provided by the myocardial ECM supports cell adhesion, proliferation, and terminal differentiation thanks to preserved biological signals and tridimensional architecture, as well as accurate removal of native cell antigens.

Taking a critical step towards the potential use of decellularized human cardiac matrix in clinical applications, composite acellular myocardium/fibrin hydrogel patches loaded with mesenchymal progenitor cells were implanted into rat models of acute and chronic myocardial infarction. Superior cell migration and angiogenesis into the infarct bed was observed in the case of acute damage, suggesting that this healing phase represents a more favourable substrate for cell-based repair than a chronic damage that has progressed to a fibrous scar [145]. A larger model of myocardial infarction was tested by implanting nanofiber-coated myocardial scaffolds injected with cord blood mononucleated cells into the ischemic myocardium of sheeps, revealing the integration of the engineered patches into the host cardiac tissue [142].

Finally, the immunogenic profile of perfusion-decellularized myocardium fragments was assessed through subcutaneous implantation in Sprague Dawley rats. While eliciting no significative inflammatory reaction, decellularized heart matrix promoted a heavy CD163^+^ macrophage response, consistent with the reconstructive remodelling of implants [141].

### 9.2. Pericardium

Decellularized pericardium has lately emerged as a promising scaffold for the fabrication of bioprosthetic cardiac valves and patches in cardiac surgery [149]. Besides providing a delivery platform to convey regenerative cells and bioactive molecules to the infarcted myocardium, the acellular pericardial matrix offers new perspectives as either a patch or an interposition graft for arterial wall reconstruction. Although xenogeneic (i.e., porcine and bovine) substitutes for autologous pericardium have been widely investigated, they still present some important limitations related to their preparation protocol. In fact, these substitutes undergo glutaraldehyde fixation, which reduces graft immunogenicity, but also causes accelerated calcification and cytotoxic effects due to the fixative remnants [149,150,151]. The solution to such problems may reside in the use of decellularized allogeneic pericardium, which would better mimic the native ECM, stimulate cellular responses and structurally/functionally integrate into the host. In this direction, many authors are working towards the decellularization of human pericardium to obtain ECM-derived scaffolds for cardiovascular TE. Human pericardial samples were obtained as discharge material from cadavers [149,150,151,152,153,154,155] or from consenting patients scheduled for cardiothoracic surgery [156,157,158,159]. Currently, various decellularization methods have been reported (Table 4), most of which are based on the use of detergent–enzymatic treatment [149,150,151,152,155,158,159]. Furthermore, recent studies successfully tested the SDS-based decellularization method [156] and a novel protocol based on the use of less traditional chemical agents (i.e., acetone and ethanol) [153,154,155]. All these processes showed a good decellularization rate and a complete removal of cell surface antigens, without affecting ECM structure. When mechanical tests were performed, a preserved resistance of the pericardial tissue to strain was generally observed after the decellularization treatment [150,151,152]. This finding supports the possible in vivo implantation inside the heart or on the great vessels, where pericardial patches will have to function in high pressure conditions. Finally, contact and extract cytotoxicity assays were carried out to assess the effect of any residual decellularization agent on cell viability and proliferation. Human fibroblasts and human epitheloid cells were successfully cultured in contact with extracts of acellular scaffolds, demonstrating the reduction of chemical/biological decellularization agents to a nontoxic level [150]. Interestingly, acellular pericardium was prepared also in the form of an injectable matrix [156] or a macroporous gel [152], by lyophilization and pepsin digestion. Moreover, Prat-Vidal and collaborators [157] tested for the first time the fabrication of a composite myocardial bioprosthesis, consisting of decellularized human pericardium embedded with a mixture of the self-assembling peptide hydrogel RAD16-I. 

Since the decellularization process might affect the ECM capacity of promoting cellular attachment and proliferation, cell repopulation studies represent a key step towards the construction of functional cardiovascular substitutes. Human dermal fibroblasts successfully attached to the mesothelial basement membrane of the human decellularized pericardial scaffold. What is more, contracture of the engineered substitute was observed two weeks after recellularization, suggesting the acquisition of contractile function by the cells in the tissue [149]. 

The fabrication of pericardium-derived 3D macroporous scaffolds enabled to attain better cellular responses in terms of proliferation, viability, migration, and differentiation of cardiac progenitor cells [152]. In addition, decellularized human pericardium was refilled with a combination of porcine mediastinal adipose tissue-derived progenitor cells and the self-assembling peptide RAD16-I. This allowed for generation of an optimal 3D network inside the scaffold, promoting cell proliferation and differentiation toward the endothelial lineage [157,158,159].

Regarding in vivo experiments, the subcutaneous implantation of decellularized pericardium into mice models demonstrated reduced cytoxicity, lowered immunogenicity, improved graft integration and lack of calcification in comparison with the fresh/frozen or glutaraldehyde-fixed implanted tissue [149,151]. Implantation of cardiac progenitor cell-loaded scaffolds into mice subcutaneous tissue was also performed to evaluate in vivo angiogenic and differentiation properties of pericardial substitutes. Ex vivo analyses on transplanted constructs revealed low immunological response, enhanced angiogenesis, and cardiomyocyte differentiation [152]. Moreover, decellularized pericardium produced as a milled matrix was injected into the left ventricular wall of healthy rats, demonstrating to form a fibrous, porous scaffold in vivo. This evidence suggests the possible use of pericardial matrix gels as structural and biochemical scaffolds to support cell infiltration and revascularization of the damaged cardiac tissue in order to control negative remodeling after myocardial infarction [156].

Chemically decellularized pericardial patches were in vivo tested into animal models of arterial wall regeneration, being implanted as a vascular patch on the abdominal aorta of rats [153,154] or the carotid/aorta of Vietnamese pigs [155]. These intravascular studies highlighted enhanced graft integration, low immunogenicity, and recellularization of scaffolds.

Finally, innovative composite pericardial ECM/hydrogel RAD16-I bioprosthesis loaded with porcine adipose-derived progenitor cells were successfully tested in vivo. The cardiac substitutes were implanted into a swine model of myocardial infarctum, over the ischemic cardiac region. Besides high biocompatibility, cell migration from the bioprosthesis to the injured myocardium was observed [157]. Moreover, the scaffold integrated with the underlying myocardium displayed recolonization, neovascularization, and nerve sprouting capability [159]. Overall, the recellularized pericardial constructs significantly reduced infarct size and improved cardiac function, reducing inflammatory response and altered collagen deposit [158].

### 9.3. Heart Valves

Among heart pathologies, end-stage heart valve disease represents an increasingly prevalent condition which can only be clinically treated by prosthetic replacement [160]. Currently, several types of heart valve substitutes are commercially available but their clinical use presents crucial drawbacks. Mechanical devices, although having higher durability, necessitate lifelong anticoagulation due to their prothrombotic nature. On the other hand, xeno- or allogeneic biologic prostheses assure excellent hemodynamic behaviour but suffer from decreased durability due to tissue deterioration [161]. In both cases, valve substitutes do not allow for somatic growth or remodeling after implantation. The search for the ideal viable valve replacement method has prompted cardiac tissue engineering to conceive biological devices with limited immunogenic and thrombogenic properties, as well as remodeling, regeneration and growth potential. Thus, the fabrication of human decellularized heart valve scaffolds seeded with autologous cells prior to implantation or recolonized in vivo after surgery represents a concept for improving current valvular heart disease therapy. In the last two decades, human heart valve allografts have been prepared by decellularization of the aortic heart valve [59,162,163,164,165], the pulmonary heart valve [57,69,166,167], or both [168,169,170,171,172]. In particular, heart valves were taken from cadavers [168], procured from a tissue banking facility [164,167,169,172], removed from the heart of the recipient during transplantation surgery [166,69], or obtained from nonheartbeating [165] and heartbeating [162] organ donors. In the perspective of engineered valve fabrication, the decellularization process plays a crucial role for its impact on ECM composition and architecture, which need to remain unchanged. According to the literature, detergents [58,61,63,65,66,67,69,163,171], enzymes [57,59,60,62,64,68,162,168,169], or both [164,165,166,167,170,172] have been used in various combinations and concentrations to achieve cell-free valve scaffolds with preserved ECM (Table 5). The marked reduction of class I and class II major histocompatibility complex expression following decellularization suggests the lowered antigenicity of valve leaflets in the perspective of in vivo implantation [57,172].

In case of heart valves, the engineered construct is required to provide sufficient mechanical function immediately upon implantation to ensure the survival of the patient. Therefore, for successful clinical applications, heart valve substitutes need to mimic and maintain the functional and mechanical properties of the native tissue. Based on that, mechanical tests were performed on acellular aortic and pulmonary valves, demonstrating that the decellularization process caused no significant impact on tissue strength or biomechanics [57,164,170,171,172]. According to extract and contact cytotoxicity tests, decellularized heart valve demonstrated to be nontoxic when cultured in contact with 3T3 murine fibroblasts and baby hamster kidney cells [172]. Interestingly, in vitro transmigration assay demonstrated that the decellularization procedure reduced the migration of human monocytes toward the heart valve tissue, suggesting a strong decrease of the humoral response during in vivo implant [166].

Despite the rapid advancement in cardiac tissue engineering research, in vitro repopulation of the acellular valve conduits remains poorly investigated. Human endothelial cells from saphenous vein [168] or the umbilical cord vein [171] were cultured under dynamic flow conditions on the luminal surface of aortic and pulmonary acellular valves, appearing as a viable confluent monolayer of cells which expressed specific endothelial lineage markers. Once seeded on acellular aortic cusp and wall, human bone marrow MSCs preserved viability in culture, confirming noncytotoxic effect of decellularization treatment [165]. Most significantly, when they were employed to repopulate decellularized pulmonary valve leaflets, human bone marrow MSCs reconstructed the endothelium lining and differentiated into fibroblasts, myofibroblasts, and smooth muscle cells. Moreover, the two valve layers—fibrosa and ventricularis—differently influenced human bone marrow MSC repopulation potential, with a higher degree of 3D spreading and differentiation in the ventricularis surface [167]. 

Finally, cardiac mesenchymal stromal cells were also seeded on acellular aortic valve matrix, adhering and proliferating onto the scaffold, as well as expressing the mature endothelial marker von Willebrandt factor. Moreover, the acquired expression of matrix metalloprotease-2 highlighted the ability of these cells to migrate from the surface to the inner layers of the leaflet structure, where a less efficient repopulation was effectively observed [162].

Taking advantage of the acellular aortic valve matrix, Koening and Colleagues [163] designed and manufactured a new decellularized and reseeded biohybrid prosthesis composed of a biological and a synthetic component. In this innovative construct, aortic valve homografts provided the cusps, while a polyurethane patch formed the walls. The biohybrid aortic valve was repopulated by the dynamic seeding of fibroblasts and endothelial cells from human saphenous vein, obtaining confluent and uniform cell layers across the complete scaffold surface [163].

Regarding preclinical studies, decellularized aortic and pulmonary valves were implanted in the subcutaneous tissue of mice, providing evidence of graft integration and recolonization by host cells [172].

In the clinical practice, decellularized aortic and pulmonary heart valve allografts have already received approval for surgical application and have been used for implant in patients with various valvular pathologies with satisfactory clinical outcomes [138,143,146,152,153,154,155] (Table 1). In particular, aortic valve allografts were succeffully tested for aortic root replacement in the presence of congenital or acquired aortic valve disease, aortic aneurysm with aortic valve disease, or native or prosthetic aortic valve endocarditis [59,63,66]. Pulmonary allografts have been employed in the replacement of the pulmonary valve for right ventricular outflow tract (RVOT) reconstruction during Ross operation [57,58,61,64] or for the treatment of pulmonary dysfunctions [57,61,62,65,67,68,69]. Remarkably, the implantation of acellular allografts did not provoke a panel reactive antibody (PRA) response, which proved to stimulate the host recellularization of the matrix and significantly improve valve and ventricular function.

To the best of our knowledge, the decellularization of mitral and tricuspid valves have been performed mainly on non-human samples [173,174,175,176,177], except for a work reported by Wan and Collaborators [178], who developed a human mitral valve-derived scaffold for cardiac repair, rather than for valvular replacement. In fact, given the difficulties in finding the appropriate human myocardial tissue, the Authors took the advantage of the higher availability of heart valve specimens which are discarded during surgery procedures. Mitral valves were harvested from patients with chordae tendinae rupture, sectioned into 50–100 µm thick slices and decellularized by SDS-based treatment. Upon seeding on acellular valve scaffolds, post-infarct murine bone marrow c-kit^+^ cells exhibited increased proliferation rate and cardiomyogenic potential in vitro. When sutured as a cardiac pach onto the epicardial surface in a murine model of myocardial infarction, the repopulated mitral valve substitute significantly improved cardiac performance and reduced infarct size.

However, ameliorating the current state of the art about the fabrication of human atrioventricular valve substitutes may represent a future research goal for the progression of heart valve regeneration strategies.

## 10. Vessels

Cardiovascular diseases, including coronary artery and peripheral vascular disease, are illnesses widespread in developed countries often requiring surgical bypass. Synthetic materials (polyethylene terephtalate fibre–Dacron; expanded polytetrafluoroethylene-ePTFE; etc.) often elicit adverse reactions due to clot development, rejection and chronic inflammation; while the availability of autologous vessels is limited [179,180]. Thus, these limits prompted towards the development of vessels with tissue engineering techniques. 

To date, different types of human vessels have been decellularized to mainly develop vascular grafts; however their use in a broad sense for tissue engineering purposes [181] or other end-use destination (i.e., cardiac patch [182]) was also considered. 

Other studies involving placental vessels are discussed in the following section ‘Placenta’.

### 10.1. Arteries

Revising the literature, many authors reported the decellularization of human umbilical cord artery (HUA) [179,182,183,184,185,186,187,188], femoral artery [189], pulmonary artery [71], internal mammary artery [190,191], arteries of the hand dorsum [15], and aortae [192,70] achieving good results in terms of structural and mechanical characteristics.

Different strategies were adopted, however the use of 3-[(3-cholamidopropyl) dimethylammonio]-1-propanesulfonate (CHAPS) and SDS was the most deeply investigated [183]. Gui and Colleagues [180] incubated HUAs in CHAPS and SDS followed by soaking in endothelial growth media-2. No significant changes in mechanical properties were found with respect to the native vessels. Rodriguez et al. [187] only used SDS and dH_2_O; while Chen et al. [188] flushed HUA with trypsin/EDTA prior to use SDS.

Mallis et al. [185] compared two protocols: (1) CHAPS and SDS followed by incubation in alpha minimal essential medium with fetal bovine serum and (2) hypotonic Tris and SDS followed by incubation in nuclease solution. Both decellularization methods proved effective in removal the cellular material, preserving the integrity of ECM; however, the first strategy was more efficient in removing DNA (6.2% versus 17.3%) and it was later adopted for further studies by the same research group [186]. 

The main advantage sought on the use of umbilical cord vessels is their possible high “on-shelf availability”; anyhow, the storage of these tissue samples even before decellularization is an important theme of discussion. To this purpose, Tuan-Mu et al. [184] considered the contribution of freezing HUAs on both decellularization and mechanical properties. Briefly, hypotonic SDS solutions were prepared with three concentrations (0.1, 0.5, and 1% (*w*/*v*)) and their effect was evaluated every 12 up to 48 h. Characterization studies proved that stiffness was not affected, but a reduction in decellularization efficiency, ascribable to ECM-condensation, was observed. 

Noteworthy, acellular patches from HUA were also prepared to improve heart function after provoking myocardium infarction in rats, by proximal left coronary ligation; in this case, the authors incubated the artery in trypsin and collagenase II mixture at 37 °C and then placed it in 10% DMEM/F12 medium [182].

Other arteries decellularization methods were based on a single freeze–thaw cycle, incubation in Tris buffer and low concentration SDS followed by treatment with DNase/RNase, as described for femoral arteries [189]; sodium lauroyl sarcosinate and a recombinant endonuclease to decellularize the pulmonary artery [71]; detergent-based methods appealing to SDS solution or Triton X-100 followed by SDS to treat mammary arteries [190,191]; dH_2_O, trypsin/EDTA, and Triton X-100/ammonium hydroxide for arteries of the hand dorsum [15]; and the aorta was processed using CHAPS, SDS, and then treated with endothelial cell growth medium-2 with fetal bovine serum [192] or SDS and DNase-I [70].

In characterization studies, the biological properties of acellular arteries were assessed by cell seeding. Working in static conditions, MSCs [182,186], CD34^+^ progenitor cells [70], human umbilical vein endothelial cells [179,188], smooth muscle cells [187] were used. Interestingly, Li et al. [182] performed cell seeding as a preliminary step to implantation; while Quint et al. [192] and Jones et al. [191] demonstrated the occurrence of an adequate graft recellularization by blood flow restoration after surgery. 

**Figure 3:**
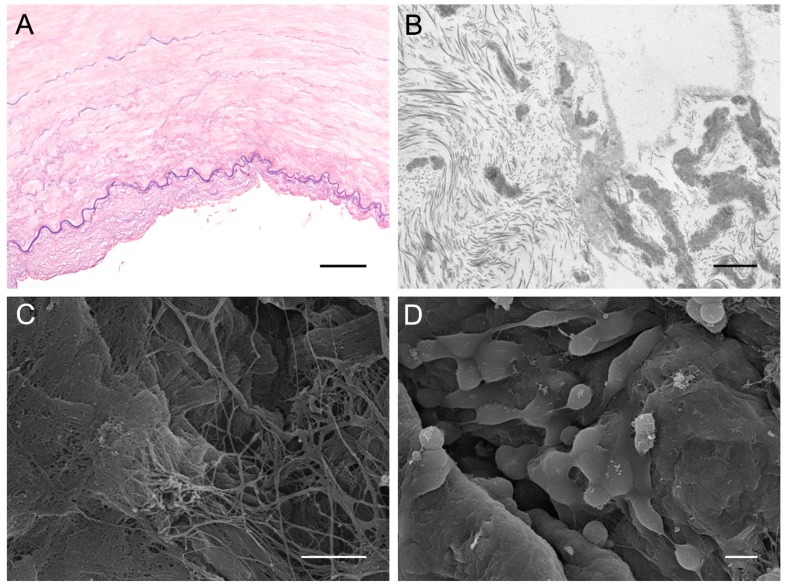
Scaffolds from human radial artery and umbilical cord Wharton’s jelly. Hematoxylin/eosin staining (**A**) and transmission electron microscope micrograph (**B**) of decellularized radial artery. Ultrastructural appearance at electron scanning microscope of Wharton’s jelly scaffold before (**C**) and 24 h after SHSY-5Y cells seeding (**D**) (unpublished data). Wharton’s jelly was decellularized, homogenated, and lyophilized according the experience of our group. Scale bars: (**A**), 100 µm; (**B**), 1 µm; (**C**), 5 µm; and (**D**), 10 µm.

Considering the experience of our group, we implanted the acellular arteries (as well as veins) in the corresponding femoral vessels of adult rabbits. After two weeks, at sacrifice, neither vessel showed signs of thrombosis or intimal hyperplasia. The arteries only showed cells at the level of adventitial and medial layers whereas partial recellularization by endothelial and smooth muscle cells was identified in veins [15] (Figure 3A,B). 

To date, a commercially available product based on allogenic pulmonary artery patch exists (i.e., MatrACELL; Life-Net Health, Inc., Virginia Beach, VA, USA). Revising the literature, two clinical trials considered its use for the reconstruction of right ventricular outflow tract [70] as well as for congenital cardiac reconstruction [71] (Table 1)—in both cases, the device did not cause adverse events or failures. Moreover, both human acellular iliac or mammary artery have been also adopted for clinical use. Two pediatric patients were implanted with these segments to treat hepatic portal vein thrombosis [72] (Table 1).

### 10.2. Veins

The researchers also considered the decellularization of veins, including umbilical cord vein, [181,183,193,194,195,196,197,198,199,200,201,202,203,204], saphenous vein [205,206], femoral vein [207], veins of the hand dorsum [15], and of amputated legs [208]. 

Beyond Hassan et al. [208] reporting decellularization by immersion in liquid nitrogen, the mainly used approaches included organic solvents, osmotic stress, ionic/non-ionic detergents and enzymes, which were experienced alone or in combination. 

Revising the literature, excised veins were processed using SDS alone [201,204,205] or SDS and ethanol with intermediate phosphate buffer saline [193] or dH_2_O rinsing [198,199]. Montoya and McFetridge [200] considered the effectiveness of a solvent mixture based on acetone/water/ethanol; in particular, the efficiency of decellularization by soaking versus a convective flow, proved that the latter was significantly more effective. A more complex protocol was later set up by Goktas et al. [194], who combined soaking phases in Triton X-100/NaCl, dH_2_O, and DNase-I to decellularize HUV-derived flat sheets.

The need to identify the optimal strategy among the many in the literature, led different groups to develop comparison studies. Uzarski et al. [195] evaluated four different protocols, highlighting that among (1) ethanol/acetone, (2) sodium chloride, (3) SDS, and (4) Triton X-100, the non-ionic detergent Triton X-100 displayed the worst results after cell seeding, maybe due to disrupted basement membrane region. Hoenicka et al. [202] studied decellularization by dehydration using a carbogen (i.e., 95% oxygen and 5% carbon dioxide) gas stream versus collagenase A and osmotic lysis by dH_2_O. The gas assured for retention of laminin, fibronectin collagen, and elastic fibres without affecting both the mechanical properties and structure of the HUV. Van de Walle et al. [196] compared SDS versus ethanol/acetone both followed by DNase-I treatment; ethanol/acetone assured for a reduction in thrombotic events with respect to SDS treated samples. Finally, Mangold et al. [197] exposed the samples to (1) Triton X-100/SDS/IGEPAL-CA630, (2) NaCl and dH_2_O, and (3) peroxyacetic acid in ethanol, prior to performing the DNase-I/RNase treatment; however, none of the above methods were adequate to support endothelial cell attachment and growth without conditioning. Recently, both the saphenous and the femoral veins were decellularized by perfusion with a method based on Triton X-100, tri-n-butyl-phosphate (TnBP), and DNase [206,207]; while Porzionato et al. [15], as previously described for arteries of the hand dorsum, also treated the veins with dH_2_O, Trypsin/EDTA and Triton X-100/ammonium hydroxide with a final dH_2_O soaking phase.

Many methods were adopted to achieve scaffolds colonization and many kind of cells were also seeded on/into flat [194,195,197] or tubular scaffolds [193,196,197,198,199,201,202,204,206,207] in static [194,195,197,198,199,201] or dynamic conditions (i.e., rotational or mechanical stimulation) [199,201,204] including perfusion [193,196,202,206,207]. It is noteworthy that some authors also considered the functional suitability of the developed supports. As early failure of bypass vessels is mainly due to thrombotic events, the potential to develop such problems was assessed in vitro by seeding platelets and/or leukocyte and evaluating their adhesion to the scaffold [195,196,204]. 

According to the literature, the cell populations considered included primary human fibroblasts and vascular smooth muscle cells [193], human gingival fibroblasts [194], and vascular endothelial cells [195]; umbilical vein endothelial cells [195,197,202], also in mixture with human cord blood-derived endothelial progenitor cells (EPC) [204], MSCs [198,199,201], and adipose-derived stem cells differentiated into endothelial-like cells, were transfected with eNOS [209]. Recellularization was also attempted by whole blood [196,206,207] with different outcomes. In fact, some authors demonstrated that the strategy is convenient and easy, as its use also avoids long cell expansion times, eventual spontaneous mutations, and discomfort for the patient [196,206]; conversely, Rambol et al. [207] did not gain good results as the method failed in veins re-endothelization.

Sometimes the end-use destination was different from cardiovascular applications (i.e., repair or bypass occluded vessels); in fact, decellularized veins were addressed for treatment of gingival recession and periodontal disease through in vitro experiments [194], peripheral nerve injuries in a animal model of damage [208], and tendons and ligaments by in vitro assays [198,199,201].

Few research papers reported the in vivo bioscaffold implantation in animal models of vascular [15,209] or other kind of damage [208].

Revising the literature, one clinical trial considered the use of allogenic acellular veins in 20 patients as a graft for hemodialysis access; none of the patients become allosensitized and no graft was lost because of infections [73] (Table 1).

## 11. Lung

Lung tissue engineering has also been proposed as possible future option to overcome the limitations of lung transplantation, such as shortage of suitable donors and rejection rates. A wide series of studies have specifically considered decellularization of human lung tissue as first step for the development of personalized lung grafts. Decellularization trials have been performed through incubation of tissue slices or fragments [210,211,212,213] and vascular and/or bronchial perfusion of lung segments/lobes or whole lung [214,215,216,217,218,219].

Recellularization of decellularized human lung tissue has also been performed though different techniques: seeding onto slices [210,212,213,216,218,220,221,222], incubation [223] or cell injection [210] of dissected segments, cell perfusion through the vasculature and/or airways of resected segments [215], whole lobes [224,225], or whole lungs [219]. Many different human cell types have been used. Primary cells include human lung fibroblasts, human umbilical vein endothelial cells, tracheal/bronchial cells, and various types of alveolar epithelial cells (reviewed in Gilpin and Wagner [226]). As it regards progenitor or stem cells, human endothelial progenitor cells [221,227], human bone marrow-derived mesenchymal stem cells [221,227], human adipose tissue-derived mesenchymal cells [227], and human induced pluripotent stem cell-derived lung progenitors [216] have also been successfully used. Alveolar cells type II derived from induced pluripotent stem cells have also been employed [210,222]. 

Approaches of decellularization and recellularization have also been performed with pathologic lungs in order to study the alterations in the ECM and its functional role in adhesion/proliferation of cells. In particular, lungs affected by idiopathic pulmonary fibrosis [228,229,230,231,232], chronic obstructive pulmonary disease [221], and scleroderma [233] were studied. To the best of our knowledge, experimental trials of surgical implantation of lung grafts derived from human ECM have not yet been performed. 

## 12. Gingiva

Revising the literature, two research articles have considered the decellularization of human gingiva [234,235] (Table 6). In particular, Mahdavishahri et al. [234] proposed the human gingival scaffold for skin regeneration purposes. Both studies preliminarly exposed the gingiva to liquid nitrogen. Naderi et al. [235] evaluated the effects of increasing SDS concentrations (0.1%, 0.5%, and 1%), observing that the decellularization efficiency increased, with reduction of GAGs content and increase in ECM porosity. Structural modifications of the scaffold due to 1% SDS decellularization method showed to adequately support rabbit blastema cells migration, proliferation and activity in vitro. Conversely, Mahdavishahri et al. [234] reported the ability of the scaffold in supporting rat bone marrow-MSCs adhesion and differentiation toward keratinocytes.

## 13. Dental Pulp

Tissue engineering is recognized as a promising approach in many fields of dentistry; however, the interest for dental pulp decellularization is quite recent. Sangkert and Collaborators [236,237] were the first authors considering decellularization of dental pulp (half-segmented teeth) by collagenase and dispase. The process succeeded in obtaining a solution that was used at a concentration of 0.1 mg/mL in 0.1% NaClO in combination with collagen or fibronectin to coat silk supports. Interestingly, an increase of scaffolds biofunctionalities was suggested by augmentation in calcium synthesis, mineralization and alkaline phosphatase activity by osteoblast-like cells (MG-63); while, ameliorated mechanical properties were proved by a higher stress value and Young’s modulus. 

Later, also Song et al. [238] considered decellularization of human dental pulp slices. The efficiency of three decellularization protocols was compared, identifying in a treatment based on hypertonic buffer + 3 cycles of 1% SDS + 1% Triton X-100 the best protocol to guarantee maximum decellularization and minimum impairment of ECM composition and organization. This finding was also proved by scaffold ability in supporting the proliferation and differentiation of human stem cells of the apical papilla.

If the works cited so far have assessed the decellularization of tooth slices, the first research article considering the decellularization of the entire dental pulp was recently published by Matoug-Elwerfelli et al. [239]. The tissue was processed according to a protocol previously described by Wilshaw et al. [240] for human amniotic membrane; it provided an ECM preserving the histoarchitecture/composition of the dental pulp and supporting human dental pulp stem cells viability and proliferation (Table 6).

## 14. Schneiderian Membrane

During open maxillary sinus lift surgery, perforations are among the most prevalent complications. To date, in clinical practice, the bone graft/substitute is isolated from the maxillary sinus cavity by the implantation of collagen membranes or decellularized scaffolds (i.e., skin subcutaneous tissues (Alloderm) and pericardium (Tutopatch)) to promote repair of the lacerated sinus mucosa [241,242].

Revising the literature, only a research paper considered the decellularization of human Schneiderian membrane (or maxillary sinus mucous membrane) to obtain a bioscaffold for tissue engineering approaches [243]. The acellular ECM was prepared by exposing the tissue to slow-freezing followed by fast-freezing and then to 1% SDS (Table 6).

Even though further studies are required, preliminary data showed that the method allowed the preservation of collagen fibers, as well as adhesion and proliferation of adipose derived MSCs.

## 15. Intestine

Significant resections of small intestine may produce the so called short bowel syndrome (SBS). The main issue related to SBS, which can also be congenital, is reduction of the absorptive surface with a consequent malabsorption condition. The high morbidity and mortality associated to SBS have prompted the research towards the identification of new treatment strategies, including tissue engineering [244]. Despite the high interest to develop a bioengineered intestine, only one research group considered human tissues to this purpose [245].

Patil and Collaborators [245] suggested the production of a tissue-engineered small intestine (TESI) combining a human intestine acellular matrix with human bone marrow MSCs. The authors experimented different decellularization methods to develop the acellular ECM but the most effective one consisted in a freezing–thawing cycle, followed by later cycles in dH_2_O, 6% dimethyl sulfoxide in hypotonic buffer, 1% Triton X-100, and DNase. Compared to treatments based on 4% sodium deoxycholate or 0.5% SDS, both followed by DNase, the preferred approach guaranteed the preservation of tissue architecture with intact villi, also mantaining both the structural proteins and some residual angiogenic factors (i.e., fibroblast growth factor-2, vascular endothelial growth factor, angiopoietin). Histological analysis of the recellularized TESI showed a gross morphology resembling the native tissue, although issues of functionality were not addressed.

Recently, Kajbafzadeh et al. [246] also worked with decellularized human small intestine but the research purpose was different, as they were looking for substitutes for bladder disorders; in fact, augmentation cystoplasty by using gastrointestinal segments is the preferred choice. Briefly, the small intestine of aborted human fetuses (gestational age <13 weeks) was decellularized by tissue perfusion with 0.5% SDS, allowing the maintenance of tissue physical properties and ECM structure. Thereafter, acellular scaffolds were also implanted in rabbits after creating a wide-mouth herniation of bladder mucosa. After four months, all typical elements of the bladder wall were recognizable as proved by histological and immunohistochemical analysis. After six months from surgery, the acellular graft was completely integrated into the host bladder.

## 16. Liver 

More than 500 million people worldwide are affected by chronic liver diseases [247]. Cirrhosis is predicted to become the twelfth leading cause of death in 2020 [248]. Transplantation of liver is the direct solution to cure and decrease liver-related mortalities. The major constraint in the implementation of transplantation programmes is the shortage of donor organs. Thus, various tissue engineering approaches have been carried out to find alternative therapies for patients suffering from liver diseases. In recent years, researchers have explored alternative uses of human livers which result to be unsuitable for transplantation. The main focus is to obtain acellular liver ECM that may be repopulated with normal liver cells in order to recreate a functional liver substitute in vitro [249]. So far, several authors have engaged in the decellularization of liver considering the whole organ [249,250], the only left lobe [249], or liver fragments [251,252,253,254]. Moreover, Baiocchini and coworkers [255] treated biopsy specimens from HCV-infected patients, aiming at correlating proteomic changes of ECM composition to the progression of liver fibrosis.

A variety of methods have been developed for the obtainment of acellular liver matrix (Table 7). When the whole organ or the left hepatic lobe was considered, the perfusion decellularization method was selected, by intravascular injection of detergents (SDS and/or Triton X-100), hyperosmotic (NaCl), and enzymatic (DNase) solutions [249,250]. On the other hand, the decellularization of liver fragments was mainly performed by immersion into detergent solutions (Triton X-100 and/or SDS) under mechanical agitation [251,253,254,255]. Finally, Mazza and Collaborators [252] standardized a new agitation–decellularization protocol, implementing the previously described detergent–enzymatic method [249] with high G-force oscillation treatment which highly reduced processing times. In general, all the described protocols assured complete removal of cellular antigens, generating hepatic scaffolds which retained the structural properties and the protein composition of acellular liver ECM. Most importantly, decellularized liver scaffolds showed to attract blood vessels and promote neoangiogenesis during a chicken egg chorioallantoic membrane (CAM) assay. Remarkably, Mattei and collaborators [251] demonstrated heterogeneous decellularization outcomes in terms of cell removal and protein/glycosaminoglycan content, highlighting the high donor-to-donor variability registered after the application of the same method. 

After the standardization of the decellularization protocol, most cited studies demonstrated that acellular ECM scaffolds can provide an optimal platform for the growth of different cell types. Whether the whole organ or small tissue biopsies were decellularized, recellularization tests have always been performed on liver ECM fragments. Seeded cell populations include a human hepatic stellate cell line (LX2) [249,252], HepG2 cells from hepatocellular carcinoma [249], and Sk-Hep-1 endothelial cells from human adenocarcinoma [249] cultured in static conditions, as well as primary human hepatocytes [252] cultured on scaffolds by a dynamic perfusion system. All the four cell types were able to engraft and migrate through the liver matrix, with high proliferation rate [249,252]. Moreover, LX2 cells colonized the sinusoidal space acquiring definite myofibroblast-like cell morphology [252]. HUVECs were also considered for liver scaffold re-endothelization, as they localize in the proximity of the decellularized blood vessels for the repopulation of the vascular line [250,252].

Finally, cell populations with stemness potential were also investigated. In particular, human induced pluripotent stem cells were seeded on decellularized liver matrix scaffolds prepared as gels and their differentiation response to the support was evaluated. Interestingly, in the presence of the only matrix stimuli, human induced pluripotent stem cells acquired specific hepatic properties which are ascribable to primary neonatal human hepatocytes [253].

The in vivo study was reported only by Mazza and Colleagues [249], who evaluated the bio-compatibility of decellularized cubic liver scaffolds in a xenotransplantation model. The samples were implanted into either the subcutaneous tissue or the omentum of immunocompetent mice up to 21 days. The xenograft elicited mild or no inflammatory reaction and promoted neovessels formation to the interface between host tissue and human scaffold, providing evidence for neovascularisation of the implant.

## 17. Pancreas

Diabetes mellitus (DM) is gaining wide attention due to its alarming increase in the rate of incidence in both adult and pediatric population worldwide. Insulin dependent DM can be managed short term by exogenous supplementation counteracting metabolic decompensation. Pancreas transplantation stabilizes the euglycemic state of the patient and thus the only reliable long-term therapy. Factors such as lack of potential donors, risk of lifelong immunosuppression, and high costs contribute to the low success rates in the transplantation regime. The need to preserve pancreas from human origin for future clinical applications remains a challenge. Regenerative therapies focusing on utilizing innate pancreas tissue is gaining momentum.

Human pancreata obtained from cadavers and unsuitable for transplantation have been decellularized as whole organs [256] or after sectioning parenchyma into approximately 1 cm^3^ pieces, as well as after tissue homogenization [257]. The decellularization methods included perfusion with Triton X-100 and NH_4_OH solution [256] or treatment with deoxycholate [257] (Table 8). In the first case, a cell free ECM scaffold, with preserved organ architecture and stiffness was obtained. In the second work, a novel ECM-based hydrogel was produced by lyophilization and pepsin/HCl digestion of the acellular pancreatic matrix for the fabrication of a fibrous 3D gel.

Regarding recellularization studies, human islet cells and endothelial cells seeded on the ECM pancreatic scaffolds survive and efficiently proliferate, providing evidence for the cytocompatibility of the repopulated supports [256]. Similarly, human pancreas-derived hydrogel (hP-HG) showed compatibility with a variety of relevant cell types including an insulinoma cell line, stem cell-derived beta-like cells, and endothelial cells, which could ultimately aid in graft vascularization [257].

In the perspective to utilize decellularized pancreas tissue for allograft TE, evaluating the induction of an immune response upon tissue substitute transplantation is of crucial importance. Sackett and coworkers [257] tested hP-HG immunogenicity by injecting a bolus of neutralized pre-gel solution into the dorsum of humanized mice and allowing it to gel in vivo. According to subsequent ex vivo analyses, the human pancreas-derived hydrogel was hypoimmunogenic, confirming that the cellular and antigenic tissue components were successfuly removed by decellularizaton treatment.

## 18. Kidney

End-stage renal disease (ESRD) is characterized by slow and progressive dysfunction of kidneys leading to atrophy and comorbidities of other organs and tissues in the body. Approximately 8–16% of the adult human population worldwide suffers from ESRD and transplantation remains the only available option of treatment [258]. There remains a wide gap between demand and supply of available organs for transplantation and the rate of incidence of graft versus host disease is increasing. Thus, there is an overwhelming need of alternative therapies to treat ESRD, taking into account the above-mentioned constraints. Tissue engineering strategies provide a valuable option in reducing the burden on traditional organ transplantation, aiming at reconstructing bioengineered renal grafts that must retain the kidney’s architecture and function. Based on this, investigating ECM scaffolds is of crucial importance in the perspective of manufacturing kidneys for transplant purposes. Thousands of kidneys are discarded each year from the total number of organs which are collected by donation-on-death programmes [259]. This has prompted researchers to investigate discarded kidneys as an allogeneic source of acellular ECM scaffolds for renal bioengineering and regeneration research [260].

Even though many researchers have successfully obtained kidney scaffolds from different species (rats, pigs, and non-human primates) [261], decellularization of human kidney has poorly been considered and remains a challenging problem.

The generation of three-dimensional acellular scaffolds has been achieved starting from whole cadaveric organs [260,261,262,263] or kidney tissue collected during nephrectomy from cancer patients, sampled in correspondence to healthy regions and cut into 2 mm-thick slices, maintaining all kidney districts [261]. Decellularization was also performed on cortex samples after removal of the renal capsule [264,265].

Human whole cadaveric kidneys were decellularized via renal artery perfusion with detergent solutions (Table 9). The detergent perfusion successfully removed resident cells and cellular debris while preserving vascular, cortical and medullary architecture, a collecting system and ureters [260,262,263]. Interestingly, renal ECM subjected to detergent perfusion demonstrated to retain the expression of growth factors (GFs) specifically involved in cell adhesion, proliferation and migration, angiogenesis, and regulation of renal functions, such as glomerular filtration [263]. The use of the detergent method was reported also for the decellularization of kidney slices and cortex fragments, obtaining completely acellular renal scaffolds while maintaining the ECM structure and composition in terms of collagen IV, laminin, and fibronectin [261,264]. Moreover, kidney cortex was furtherly processed by lyophilization and homogenization to fabricate a novel ECM hydrogel [264].

Reported in vitro characterization of decellularized human kidney included resin casting and pulse-wave measurements to verify that ECM preserves microvascular morphology and morphometry and physiological function [263]. In addition, an interesting study consisted of performing chicken chorioallantoic membrane (CAM) assay, which confirmed the proangiogenic activity of renal acellular ECM [260].

Reviewing the literature, very few information can be found about recellularization of human decellularized kidney matrix. So, the identification of the ideal cell types which can successfully functionalize human renal scaffolds still remains a crucial challenge. To generate functional tissue, Bombelli and colleagues [261] proposed nephrosphere (NS) cells, composed by renal stem/progenitor-like cells, as an ideal source for renal scaffold repopulation. These cells were seeded on acellular renal slices with or without the addition of epithelial/endothelial soluble stimuli. They were able to repopulate different nephron portions of the decellularized scaffold, including cortex, medulla, and papilla. Moreover, in the absence of specific differentiation factors, they responded to ECM induction stimuli of the ECM, generating both tubular and endothelial-like structures. When specifically stimulated, they were induced towards only a specific differentiation—epithelial or endothelial—lineage [261].

An in vitro model of kidney peritubular microvasculature was created by Nagao and Collaborators [264] by seeding cortex ECM hydrogels with human kidney peritubular microvascular endothelial cells, in comparison with human umbilical vein endothelial cells. Cultured on kidney scaffolds, the first cell population became more quiescent, whereas the second one became more stimulated. Moreover, when incorporated into an engineered 3D microvascular network and seeded with peritubular microvascular endothelial cells, the kidney ECM gel demonstrated its supportive function for cell proliferation.

In conclusion, the lack of in vivo implants of engineered human renal constructs represents a significative gap in knowledge.

## 19. Bladder

Various clinical conditions such as strictures, traumatic/congenital defects, and cancer result in poor urethral/bladder compliance, requiring tissue augmentation, or substitution. Since organ-specific mucosa is often lacking to fulfill adequate reconstruction criteria, different alternative sources have been evaluated, including genital and extragenital skin flaps or grafts, mucosal grafts from buccal regions, tunica vaginalis, peritoneal grafts, and small intestine submucosa [266]. Nevertheless, the most preferable material for urethral/bladder reconstruction still remains the bladder acellular matrix, which may be collected from cadaveric donors to address the shortage of homologous tissue available for human transplant.

At present, most of the studies found in the literature deal with the investigation of rat, porcine and canine bladder matrix for tissue engineering purposes [266,267]. On the other hand, decellularization of human bladder collected from cadavers or during surgery was poorly considered. A first protocol used 10 M PBS and 0.1–1% sodium azide to allow partial cell lysis, followed by 1 M sodium chloride containing 2000 KU deoxyribonuclease to complete cell lysis with the release of all cellular components. Finally, the solubilization of the lipid bilayer cell membrane and intracellular membrane lipids was obtained by treatment with 4% sodium deoxycholate containing 0.1% sodium azide [268,269]. An alternative decellularization method was successfully standardized to produce a bladder submucosal collagen-based inert matrix to be used as a homologous graft for urethral/bladder repair [74,75,76,77]. Briefly, the bladder submucosa was microdissected and isolated from the adjacent muscular and serosal layers and then treated with distilled water, followed by 0.5% Triton-X 100 and 0.05% ammonium hydroxide. Overall, both the reported protocols assured complete cell removal from the matrix, with preservation of elastic and collagen fibres.

In vitro investigation of decellularized bladder matrix include the assessment of biomechanical properties, highlighting no significant differences for strength, strain or elastic modulus between the acellular bladder tissue and the native counterpart [268].

Regarding recellularization experiments, the acellular bladder matrix—whether alone or combined with polyglycolic acid—was engineered by seeding of autologous urothelial and muscle cells. More precisely, the exterior surface of the bioscaffold was seeded with the smooth muscle cells, whereas the internal surface was coated with urothelial cells, in order to recreate the original tissue structure [76] (Table 1).

Furthermore, a different group of authors reported the in vivo implant of decellularized human bladder both in animals and in human recipients. In particular, Sievert and collaborators [269] compared the in vivo regeneration capacity of homologous (canine) and heterologous (human and monkey) acellular bladder matrices implanted into dogs after partial cystectomy to remove 50% of the native bladder dome. Seven months from surgery, implanted animals exhibited higher bladder capacity than untreated controls, regardless of graft origin. However, the histologic examination revealed that the homologous grafted tissue assured the best regeneration performance, with a faster and more complete smooth muscle cell colonization of the scaffold. Nevertheless, the heterologous matrix did not elicit any rejection reaction, assuring for complete internal epithelialization and angiogenesis of the graft [269].

Remarkably, the clinical efficacy of a human bladder submucosal collagen-based inert matrix for urethral reconstruction was investigated by graft implantation into patients with hypospadias [74] and urethral strictures [75,77] (Table 1). The clinical studies demonstrated that the use of acellular bladder matrix achieved satisfactory results in terms of urethra patency, voiding improvement and neoformation of the typical urethral stratified epithelium, offering a promising option for urethral repair. In addition, engineered homologous bladder and bladder/polyglycolic acid scaffolds seeded with autologous urothelial and muscle cells were implanted into patients needing cystoplasty due to end-stage bladder disease [74]. The grafts demonstrated positive clinical outcomes, providing for adequate phenotypical, structural and functional recovery of bladder tissue.

## 20. Male Reproductive System

Tissue engineering of human testis has also been proposed for prepubertal boys facing cancer therapies showing gonadotoxicity. In these cases, immature testicular tissue containing spermatogonial stem cells can be cryopreserved. In adulthood, fertility could be potentially restored through transplantation of the tissue itself or its spermatogonial stem cells, or through in vitro maturation [270]. Although good results have been obtained in animals, clinical trials have not yet been performed. In particular, the transplantation of unselected testicular cell suspensions or tissue is not possible due to the risk of reintroducing neoplastic cells in hematological cancers. Thus, tissue-engineered testis, obtained from acellular testis scaffolds and isolated spermatogonial stem cells, could be useful to achieve spermatogenesis in vitro or for tissue grafting in vivo [271]. Human cadaveric testes have been decellularized by Baert et al. [272] with different methods: 1% Triton X-100 (24 or 48 h), 1% SDS (24 or 48 h), or serial treatment with 1% Triton X-100 (24 h) and 1% SDS (24 h)—twenty-four hours of 1% SDS was the best protocol. Cell attachment and integration was studied through seeding of human testicular cells obtained from bilateral orchidectomy due to prostate cancer. Seeded cells attached to the acellular scaffold surface and partially infiltrated the matrix, although they were unable to recreate structures similar to seminiferous tubules [273] (Table 10). Some authors have proposed the use of pig immature testicular tissue to obtain acellular scaffolds for testis tissue engineering, in order to avoid the use of human material. The lack of cytotoxic effect of porcine scaffolds on human Sertoli cells was demonstrated by using an indirect contact culture system or by directly seeding cells onto the supports [271].

Congenital anomalies, traumas or tissue loss due to malignancies may need surgical reconstructive approaches to the male external genitals. Approaches of tissue engineering and regenerative medicine have recently been proposed in order to prevent donor-site morbidity due to flap grafting procedures. Decellularized tunica albuginea of the human penis has been proposed for reconstructive procedures of penile curvatures; two different decellularization protocols (PEG 1000 and Triton X-100) proved efficient in preserving structural and biochemical properties [274] (Table 10). In the view of complex reconstructive surgery, human glans tissues obtained from donors for transplantation have been decellularized through perfusion into the deep dorsal vein of the penis of a solution with 1% Triton X-100 and 0.1% ammonium hydroxide [275]. In vitro recellularization was also performed through seeding of a cell suspension of rat mesenchymal stem cells, good viability and integration was achieved after 7 and 14 days but not after 28 days. Corpus cavernosum tissues have been successfully decellularized through repeated/alternated perfusion cycles of Triton X-100 and SDS into the body of tissue [276]. Corpora cavernosa were obtained from sex reassignment surgery. In vivo recellularization was performed through transplantation of a section of decellularized scaffold into the omentum of adult rats and location into the scrotum; good endothelial and smooth muscle cell recellularization was achieved after six months. An analogous approach was also followed by the same research group for decellularization and in vivo recellularization of human urethra surrounded by its corpus spongiosum [277]. In this second work, however, in vitro recellularization was also performed with mesenchymal stem cells for preputial tissue, through static-based or perfusion-based procedures. Although all procedures gave good results, in vivo recellularization proved superior to in vitro techniques and cell sheet engineering was more satisfactory than perfusion-based method. 

Prostate decellularization has not been proposed for tissue engineering purpose. However, Cazzaniga et al. [278] developed an ex vivo/in vitro model involving decellularization of prostate specimens from radical prostatectomy and following co-culture with primary prostate cancer cells. This model proved reliable to evaluate the invasiveness of different benign or malignant cell lines. 

## 21. Female Reproductive System

The development of engineered ovarian implants has also been proposed for patients affected by ovarian insufficiency due to therapy-induced ovarian failure (cancer or rheumatologic disease), or autoimmune or genetic disorders. Hassanpour et al. [279] recently decellularized human ovaries in order to obtain human acellular scaffolds potentially retaining the specialized characteristics of the microenvironment (including growth factors and cytokines) for proliferation, survival, and steroidogenesis. Efficient decellularization was achieved both with ovarian pieces (1% sodium lauryl ester sulfate-SLES for 48 h and DNase I for 24 h) and whole organ (1% SLES for 30–40 days). Preliminary cytocompatibility test with human Wharton’s jelly mesenchymal stem cells showed good cellular viability and proliferation. Human acellular scaffolds were also seeded with rat primary ovarian cells and implanted into immature ovariectomized rats, producing serum estradiol and progesterone levels and vaginal patency. Histological analyses confirmed the presence of a high number of viable ovarian cells, expressing estradiol- and progesterone-receptors and organized in follicle structures. 

Congenital or acquired structural defects in the uterus have also been proposed to be treated with neo-myometrial tissue patches. Young and Goloman [280] developed human acellular scaffolds through decellularization of myometrial biopsies obtained during Cesarean deliveries. Recellularization was also performed with seeding of human myometrial cell lines. However, better results were paradoxically reported with human myometrial cells on scaffolds obtained from rat myometrium, where the surface multicellular layers were thicker, clusters of myometrial cells were identifiable in depth and coordinated contractions were found at isometric contractility tests. 

In order to develop a new experimental tool for studying endometrial biology, human endometrial samples have been decellularized and recellularized through seeding of primary culture of human endometrial cells. Tissues were obtained from premenopausal women undergoing hysterectomy and decellularization protocol involved a mixture of 0.25% Triton X-100 and 0.25% SDS, followed by ribonuclease and DNase I [281]. Although the study was not aimed to tissue engineering approaches, its results may be useful for future development of full thickness endometrial–myometrial grafts (Table 10). 

## 22. Products of Childbirth: Umbilical Cord, Placenta and Amniotic Membrane

The umbilical cord, placenta, and amnion are all considered waste products of delivery. 

Because they are inexpensive, easily available, and without ethical concerns [197], these tissues are ideal candidates as biomaterials for tissue engineering [240]. 

### 22.1. Umbilical Cord (Wharton’s Jelly)

In addition to the vessels (see above in Vascular grafts paragraph), the umbilical cord shows a firm mucoid connective tissue called Wharton’s jelly (W’sJ) which started to be considered for tissue engineering applications quite recently due to its high content in collagen, fibronectin, and growth factors [282].

To our knowledge, the first attempt to decellularize human W’sJ was developed by our group [36]. We adopted a method based on a detergent enzymatic protocol that provided an acellular ECM conserving collagen, mucus, and reticular fibres but with low fibrinoid elements (Figure 3C,D) (Table 11). Thereafter, other three research papers involved human W’sJ use for tissue engineering [283,284,285,286,287]. Even though different decellularization protocols were applied, each of them assured a good outcome as proved by characterization studies or in vitro/in vivo assays. In fact, Jadalannagari et al. [283] obtained an acellular ECM rich in GAGs, hyaluronic acid, collagen (types II, VI, and XII), fibronectin-I, and lumican I as assessed by mass spectrometry. According to histological and biochemical analysis, Beiki et al. [284] employed a decellularization method which guaranteeded the manteinance of bioactive molecules as well as a highly interconnected porous structure.

Regarding in vitro studies, W’sJ acellular ECMs (combined with a synthetic polymer after homogenization and lyophilization; as a spongy scaffold, after homogenation, lyophilization, and crossliking; and as a wafer, after OCT—Optimal Cutting Temperature compound—embedding and cut) supported adhesion of cell lines (human fibroblasts cell line—HSF-PI 18; leukemia cell lines—HL-60, Kasumi I, and MV 411), primary cells (human chondrocytes), and stem cells (from Wharton’s jelly, bone marrow, and umbilical cord blood) [36,283,284,285,286,287]. Interestingly, after penetrating the scaffold, MSCs reduced their proliferation, as proved by the decreased expression of the specific genes (i.e., MKI67—antigen identified by monoclonal antibody Ki-67 and PCNA—proliferating cell nuclear antigen); moreover, no clear pattern of differentiation was observed, suggesting the need for further analysis [283]. 

Considering in vivo studies, the implantation of both unseeded and seeded W’sJ scaffolds addressed different tissue-defects. Beiki et al. [284] studied the potential of decellularized W’sJ to regenerate skin in mice in case of full-thickness wounds. After one week, the scaffolds were well integrated into the tissue and the wound was completely closed with disappearance of the scab; complete re-epithelialization occurred after 12 days. W’sJ-derived wafers, previously seeded with labeled osteocytes (i.e., green fluorescent protein labeling), were also implanted in a murine calvarial defect model showing the ability to produce ECM up to 14 days after transplantation [283] (Table 11).

### 22.2. Placenta

Many authors have described the decellularization of human placenta for tissue engineering applications (Table 12). Researchers’ interest towards this tissue is related to its high content in ECM components and well-preserved endogenous growth factors [286]. To our knowledge, the first experimental work was developed by Flynn et al. [287,288], considering its potential function as an adipose tissue substitute in plastic surgery for reconstructive, corrective, and cosmetic procedures. Preserving the vascular network of the tissue, large segments (8 × 8 cm) were entirely decellularized by a perfusive and diffusive decellularization protocol involving treatment with hypotonic and hypertonic solutions, enzymatic digestion, and multiple detergent extractions for a total amount of 18 days. Interestingly, the Authors also reported their previous experience with different solutions to decellularize placenta, highlighting that the non-ionic detergent Triton-X100 alone was not effective to fully decellularize large tissue samples. On the other hand, using SDS may result in a compromised and not completely decellularized ECM with also drawbacks in vitro and in vivo due to the presence of entrapped residues of the chemical agent in the tissue. Regarding lauroyl sarcosinate, despite good results in decellularizing placenta, it appeared to interfere with the enzymatic digestion of the residual DNA and RNA of the matrix; hence, the authors developed the decellularization protocol also considering this aspect. Thereafter, primary human adipose precursor cells were seeded on the scaffold which showed to support cellular adhesion at early time points (24 and 72 h) but, by the 7-day time point, the seeded cells were nonviable, maybe due to the complex architecture of the matrix responsible for their dispersion. 

Choi et al. [289] considered the potential role of human placenta as a dermal substitute for full-thickness wound healing also by virtue of its biological properties (anti-inflammatory, antibacterial, low immunogenicity, antiscarring, and wound protection). Unlike other protocols, here ECM was extracted by tissue homogenization and then decellularized through physical, chemical, and enzymatic treatments. Thereafter, porous sheets of ECM were prepared through molding and freeze-drying. The efficacy of the ECM-sheets was tested and proved in vivo by implantation in a full-thickness circle wound created in the upper back area of rats.

The scaffolds showed to promote the wound closure producing a dermal substitute with a cellular organization very similar to that of normal skin with epidermis and dermis restoration in 4 weeks, hair follicles and microvessels. 

The use of human decellularized placenta for growth of organs and tissues in vitro and in vivo was experimented by Kakabadze and Kakabadze [290] and Kakabadze et al. [291]. The authors supported the idea that the angioarchitechtonic structure of the tissue may provide adequate nutrition of transplanted cells and tissues. Decellularization occurred through perfusion via placental arteries; SDS and Triton X-100 were both used. Thereafter, the development of hepatized placenta occurred by injecting up to 150 mL-liver fragments, 10–15 mL per cotyledon, through the placenta capsule. The study also proved the high mechanical strength of decellularized placental vessels along with the capacity to bridge arterial and venous defects, suggesting their potential use for vascular reconstruction or bypasses.

In 2017, Francis et al. [292] evaluated the potential of a solubilized human placental plate tissue-derived ECM preparation for cardiac cell culture in vitro, as well as its therapeutic application as a cell-free treatment in case of post myocardial infarction in vivo. The hydrogel proved to be able to support cardiomyocytes and reduced scar formation while maintaining electrophysiological activity when injected into ischemic myocardium of a rat acute myocardial infarction model. 

Schneider et al. [9,25] focused on the decellularization of vessel grafts from human placental chorionic plate. In their first work [25], the authors preferred Triton X-100 to SDS, reporting that SDS-related protein denaturation and structural alterations may have an impact on mechanical properties and biocompatibility of the scaffold. The authors also sustained that perfusion enabled to use lower concentrations and shorter exposure times compared to nonperfusion processes. Scaffolds were then efficiently recellularized with primary human endothelial cells. In the latter study [9], they also obtained small diameter vascular grafts from human placenta through two different decellularization methods, based on Triton X-100 or SDS, also involving cross-linking with heparin. The grafts were cultured with primary human macrophages and then subcutaneously implanted in nude rats, showing good results in biocompatibility. Moreover, good graft performance was demonstrated using an aortic implantation model in Sprague Dawley rats. 

### 22.3. Amniotic Membrane

As it was demonstrated by many clinical cases and animal studies [293], the efficacy of amniotic membrane in promoting soft tissues healing and regeneration is due to its anti-inflammatory and immunological properties as well as to cytoprotective ability [120,121,122,123,124,125,126,127,128,129,130,131,132,133,134,135,136,137,138,139,140,141,142,143,144,145,146,147,148,149,150,151,152,153,154,155,156,157,158,159,160,161,162,163,164,165,166,167,168,169,170,171,172,173,174,175,176,177,178,179,180,181,182,183,184,185,186,187,188,189,190,191,192,193,194,195,196,197,198,199,200,201,202,203,204,205,206,207,208,209,210,211,212,213,214,215,216,217,218,219,220,221,222,223,224,225,226,227,228,229,230,231,232,233,234,235,236,237,238,239,240,241,242,243,244,245,246,247,248,249,250,251,252,253,254,255,256,257,258,259,260,261,262,263,264,265,266,267,268,269,270,271,272,273,274,275,276,277,278,279,280,281,282,283,284,285,286,287,288,289,290,291]. Even though commercially available decellularized human amniotic membranes exist (i.e., Acelagraft™ and Biovance™, Celgene Cellular Therapeutics, Morris, New Jersey) [78,294,295], a careful revision of the literature highlighted that, since 2006, the interest toward this tissue has significantly increased to address many different tissue healings according to tissue engineering principles. 

In particular, acellular amniotic membrane was investigated as a scaffold to support/deliver epithelial cells [240], limbal stem cells [296], human adipose stem cells [297], differentiated neural-like cells [298], mice ovarian follicular culture [299], as well as a scaffold with cell-guiding ability [300] but it may also be used as a surgical patch/mesh [301] for reconstruction of vessels [302] (also once rolled up [303,304]); bladder [305] or circumferential urethral defect [306], reconstruction of esophageal wall [307], cardiac [120,308] and cerebrospinal fluid [308] applications, endometrial fibrosis treatment [309], soft tissue damage, limbal stem cell deficiency [293], skin defects also due to ulcers or severe skin burns or for corneal defects [310,311,312], treatment of pharyngocutaneous fistula after total laryngectomy [291], and for fetoscopic closure of iatrogenic defects in fetal membranes [313]. 

Several decellularization methods were approached, often combining multiple phases based on physical (freezing–thawing), osmotic-chemical (Triton X-100, SDS, EDTA, Tris, NaOH, NH_4_Cl) and/or enzymatic (trypsin, dispase, lipase, DNase, and RNase) treatments [120,234,289,291,292,293,294,296,297,298,299,300,301,302,303,304,305,306,307,308,309,311,312,313,314,315,317,318,319]. 

Acellular human amniotic membrane also demonstrated to support different types of stem and nonstem cells. Stem/progenitor cells considered for recellularization were the following: rat bone marrow MSCs, differentiated toward neural [308], adipogenic and osteogenic lineages [314]; rabbit limbal stem cells [293]; human epicardial progenitor cells [144]; adipose derived MSCs [297,312]; human amniotic [298,313]; Wharton´s jelly MSCs, differentiated into neural-like cells [298]; rat bone marrow stromal cells [315]; human telomerase-immortalized corneal epithelial cells (tHCEC), primary limbal epithelial stem cells (LESC), LESC-derived human induced pluripotent stem cells, or skin fibroblast-derived human induced pluripotent stem cells [296]; thymus and cord-blood-derived MSCs [302]; bone marrow MSCs; and EPCs from canine new stillbirth bone marrow [306].

Various nonstem cells were also investiganted and showed good repopulation properties: rabbit macrophages [293]; murine HL-1 cells and human immune cells derived from buffy coat [144]; mice primary follicles [299]; human keratinocytes [311]; porcine oral epithelial cells [307]; human amniotic epithelial cells [298,313]; fibroblasts (mouse embryonic—NIH3T3; human cardiac, foreskin, dermal, and nasal turbinate) [144,311,313,316,317,318]; rabbit, mouse, and human smooth muscle cells [301,305]; cardiac myocytes, [302]; human skeletal muscle cells [300]; human endothelial cells [302,303,313]; human chondrocytes [313].

The in vivo behavior of AM-derived scaffolds was evaluated in: rat model of intrauterine adhesion [306]; third-degree skin burns in BALB/c mice [312]; full-thickness skin defects in Sprague Dawley rats [310]; patients experiencing pharyngocutaneous fistula because of surgical treatment of laryngeal squamous cell carcinoma [291]; BALB/c mice in which myocardial infarction was induced [319]; uterus of rabbits [301]; subcutaneously in the left hemiback of rats [316]; in the left pulmonary artery of pig after a left posterolateral thoracotomy [302]; in dogs urethral defect model [306]; for closure of fetoscopic entry wounds in the exposed rabbit amniotic sac [313]; and deep flexor tendon of the chicken toe [320].

Interestingly, some authors also considered the development of more complex decellularized amniotic membrane scaffold as they combined the acellular tissue with electrospun polycaprolactone, poly (lactic acid), and poly(d,l-lactide-co-glycolide [293]; with human myocardium ECM [144]; with electrospun nanofibrous silk fibroin [312]; with poly(ester urethane) [301]; and with aligned electrospun fibers of PLGA [300]. Also amniotic membrane-based multilayered scaffolds were manufactured [302].

Revising the literature, a commercial product has been developed from human acellular amniotic membrane (i.e., Biovance—Celgene Cellular Therapeutics, Morris, NJ, USA). In 2009, Letendre et al. [78] developed an open-label study of 14 patients with chronic nonhealing diabetic partial- or full-thickness ulcers. According to the results, at 12 weeks, 60.1% of total participants had a benefit from using Biovance wound covering; moreover, no adverse reaction to the tissue was observed (Table 1).

## 23. Cornea

Corneal damages (i.e., genetic diseases, ocular burns, and trauma to the eye) often lead to irreversible opacity of the cornea, which is estimated to be the fourth leading cause of blindness globally according to World Health Organization, 2015. The current clinical treatment is mainly based on the replacement of the damaged or diseased cornea with donated cadaveric corneal tissue [321]. The surgical procedure consists of replacing the entire cornea (penetrating keratoplasty, PK) or only the injured layer component (lamellar keratoplasty). In particular, Descemet’s stripping and endothelial kerotoplasty (DSEK) is turning out to be the preferred strategy when the corneal stroma is not scarred. It has been documented that this strategy promotes accurate vision restoration more rapidly and with fewer complications in comparison to traditional PK [322]. Although they have been accepted as standard procedures, these corneal transplantation methods still suffer from some important limitations [323] mainly due to short availability of quality-donor graft material, and occurrence of graft failure caused by immunological responses to epithelial or endothelial antigens [323,324]. Corneal tissue engineering is proving to be a promising answer to overcome this problem. The cornea represents an ideal tissue to be engineered because it contains relatively few cell types. Furthermore, it is an avascular tissue that is less exposed to the immune system, which lowers the probabilities of graft rejection. On the other hand, the cornea has many unique challenges, since it needs to be transparent and appropriately shaped to transmit and refract light [325,326]. In recent years, decellularized corneas have been investigated as scaffolds for corneal tissue engineering (Table 13). The main tissue source was cadaveric donor corneas which were unsuitable for transplantation because of low cell counts, positive serology or nonviable epithelium/endothelium. Besides processing the entire cornea [324,326,327,328] for native cell removal, some authors described the decellularization of the full tissue or the only corneal stroma after slicing them into 90–200 µm thickness sheets by a microtome or cryotome [322,329] or cutting them by femtosecond laser [330] for the obtainment of multiple thin corneal lamellae. Most recently, some authors [331,332] considered to recycle corneal stromal lenticules derived from small incision lenticule extraction (SMILE), which is rapidly spreading as a novel femtosecond laser-based procedure to correct myopia. 

While some works have focused on the evaluation of a single decellularization method, other studies have reported the comparison of different protocols borrowed from the literature, aiming to find an appropriate balance between removal of cellular elements and preservation of corneal structure and functionality. In summary, the following decellularization methods were tested. (a) The detergent method, by the use of SDS or Triton X-100 [322,324,326,327,331]; (b) the treatment with ethylenediaminetetraacetic acid (EDTA) followed by mechanical abrasion of the epithelium [328]; (c) the incubation with enzymatic solutions [324,331]; (d) the detergent–enzymatic treatment [79,326,329]; (e) the enzymatic disaggregation using dispase [324]; (f) the hyperosmotic method with sodium chloride (NaCl) [324,331]; (g) the NaCl + nucleases (DNase/RNase) method [326,327,332]; (h) the osmotic gradient + detergent method, consisting of treating corneas with a combination of hypotonic and hypertonic buffers along with Triton X-100 and SDS [327]; (i) the poly(ethylene glycol) (PEG) method, which avoids using detergents, taking advantage of this amphiphilic polymer to damage the cell membranes [327]; (j) the liquid nitrogen method, based on freezing the tissues and maintaining them in a hypoxic environment [327,330]; (k) freezing/thawing [330,333]; (l) mechanical agitation [324,330]; and (m) detergent treatment with SDS +/− DNase + mechanical agitation [330]. In general, combining detergent and enzymatic treatments seemed to provide the most superior results, assuring complete cell removal, preservation of the native ECM components (collagen type I, II, III, and IV, fibronectin) and maintenance of the biomechanical/optical properties functional to corneal transplantation.

Most of the reviewed works investigated the structural integrity of decellularized corneas and possible cytotoxic effect of the chemical remnants from the process by a cell line proliferation assay. Acellular corneal scaffolds were successfully repopulated by human corneal endothelial cells (CECs) [322,326,330], human corneal epithelial cells [327] and fibroblasts [327,331], human adipose derived adult stem cells (h-ADASC) [329], and human limbal epithelial cells (hLEC) [326,328]. 

Instead of scaffold recellularization, Wilson and colleagues [324] investigated the biocompatibility of the decellularized corneas by co-culturing human corneal stromal cells (CSCs) with small pieces of acellular corneal matrix or native corneal tissue placed onto cell culture insert dishes. Cell proliferation assay demonstrated that cell growth was significantly reduced in the presence of decellularized tissues, probably due to compromised structure and diminished GAG content. The authors concluded that the possibility of recycling unsuitable donor corneal tissue for clinical translation depends on the need to find a decellularization criterion which should balance effective removal of immune molecules and preservation of tissue architecture/functionality [324].

Interestingly, He and collaborators [330] tested acellular corneal lamellae repopulated with human corneal endothelial cells for the surgical handling by creating an endothelial graft in vitro model. Briefly, recellularized lamellae were injected into the anterior chamber of a freshly enucleated cadaveric eye ball. 

The in vivo surgical implant represents the final validation of the engineered corneal substitutes. Three works reported the implantation of neo-corneas into a rabbit corneal stroma, confirming the safety and biocompatibility of decellularized scaffolds based on the absence of short- or long-term immune reactions [329,331,332]. Moreover, Bhogal and colleagues [333] removed the Descemet’s membrane from the decellularized cornea and implanted it into the corneal endothelium of rabbits after creating a peel wound by descemetorhexis. Acellular Descemet’s membrane transplantation recovered the abnormal peel phenotype restoring corneal thickness and transparency.

Recently, the safety and efficacy of decellularized human corneal transplantation for thickness recovery of advanced keratoconic eyes have been preliminary evaluated in a phase 1 clinical trial [79]. Corneal stromal laminas were decellularized according to Alió del Barrio and Colleagues [329] and recellularized with autologous adipose-derived adult stem cells. Six months after implantation all patients presented no clinical haze or scarring, showing a general improvement of all visual parameters (Table 1).

## 24. Vocal Folds

The structure of the ECM of vocal folds is extremely important in phonation; hence, in the case of conditions compromising them, tissue engineering approaches could be helpful [334]. To date, studies regarding the decellularization of human vocal folds are scant and they mainly regard the proteomic characterization [335] or the microstructural analysis [336] of the acellular tissue. Only Li et al. [337] also considered recellularization, highlighting the significant role of phenotypically adequate cells in scaffold remdeling.

Different decellularization protocols were experienced: Welham et al. [335] exposed the tissue to repeated cycles of osmotic stress by NaCl followed by nuclease treatment (DNase I and RNase A) up to a dehydration phase in 70% ethanol. The decellularization required six days. Thereafter, the authors performed a mass spectrometry-based proteomic analysis to characterize the ECM; interestingly, they were able not only to fully characterize the matrix but also to recognize the existence of unidentified bioactive molecules that may favour tissue regeneration and remodeling, or determine reactions like chronic inflammation, scaffold encapsulation, and fibrosis, thus influencing host response after the implantation. Conversely, Tse and Long [336] took advantage from a treatment with only 0.1% SDS in deionized water. The characterization study of the acellular matrix proved the efficiency of the method, as the mechanical properties were not significantly compromised. Moreover, even if hyaluronic acid was lost, contents in collagen, elastin, and laminin were maintained. Neither of the studies considered in vitro recellularization or in vivo implant approaches. 

As regards Li and Collegues [337], five decellularization protocols were tested and compared. Tissues were treted with the following methods, (1) 1% CHAPS; (2) 1% SDS; (3) 3 M NaCl, DNase I/RNase A, 70% ethanol, DNase I/RNase A; (4) Method 3 followed by Method 1; (5) Method 3 followed by Method 2. By the end, Method 4 was chosen as the most proper one. Recellularization was performed with a vocal fold-derived fibroblast cell line; thereafter, proteomic analysis was done by a stable isotope labeling with amino acids in cell culture (SILAC)-based method. This approach allowed to distinguish constitutive proteins of the acellular scaffolds from the ones newly synthesized revealing that host cells remodeled the scaffold promoting ECM turnover.

## 25. Peripheral Nerves

The gold standard for peripheral nerve injuries with gap is bridging the stumps using autologous nerve grafts but this approach may be difficult due to donor-site morbidities, size mismatch, poor functional recovery rates, and longer surgery duration. Other options include bridging the gap using natural or synthetic nerve conduits [338], but the ideal method to guarantee a full recovery of the damaged nerve is still lacking; hence tissue engineering may be encountered as an interesting approach. As reviewed by Szynkaruk et al. [339] researchers have long been interested in setting up nonimmunogenic peripheral nerves allografts. In particular, working on murine sciatic nerves or peroneal nerves, they considered different protocols taking advantage from cold preservation [340], freeze thawing [341,342], detergents [343,344], or irradiation [345,346]. Despite complexity, only the detergent protocols proved to be effective, in accordance with the first findings by Johnson et al. [347] who developed one of the most common chemical-based decellularization methods (later refined by Sondell et al. [348]), working on human peripheral nerves (i.e., lumbar plexus, posterior tibial nerve, radial nerve, posterior cord of the brachial plexus, sural nerve, and lumbar dorsal root ganglia). 

To date, there exists only one commercial product based on acellular peripheral human nerve that is Avance^®^ (AxoGen, Inc., Alachua, FL, USA). Avance^®^ is a processed human allograft produced by the the detergent method developed by Hudson et al. [344] plus a chondroitinase treatment. Briefly, the protocol occurs at 25 °C with agitation, and provides for exposure of the tissue to: solution with 125 mM sulfobetaine (SB)-10, 10 mM phosphate and 50 mM sodium; washing solution based on 50 mM phosphate and 100 mM sodium; 0.14% Triton X-200, 0.6 mM SB-16, 10 mM phosphate, and 50 mM sodium; washing solution 3×; SB-10 solution; SB-16/Triton X-200 solution; wash 3X in 10 mM phosphate, 50 mM sodium solution. A treatment with chondroitinase ABC was also performed to remove regeneration-inhibiting chondroitin-6-sulfate [349]. 

Whitlock and Colleagues [349] used Avance^®^ to repair the transected sciatic nerve of rats in comparison with the type 1 collagen conduit NeuraGen^®^ (Integra, Plainsboro, NJ, USA) and isograft; similar results among the experimental groups were observed only in the occurrence of a short gap model (14 mm) but not in a long one (28 mm), where isografts or decelluarized allografts were preferable [350,351]. 

Regarding clinical efficacy studies, only a case report study is reported in the literature. In 2010 Shanti and Ziccardi [352] considered Avance^®^ for the reconstruction of a segmental inferior alveolar nerve defect in a 62 years old woman, highlighting its potential for peripheral trigeminal nerve reconstruction in the presence of large nerve gaps. Unfortunately, studies on this end-use of Avance^®^ are lacking; hence, increasing research would be an important object of interest especially for clinical practice.

## 26. Complex Composite Structures

Decellularization of complex structures made up of many different tissue types has also been proposed. Gerli et al. [353] recently reported the technical feasibility of perfusion decellularization of a whole human upper limb sampled from a cadaver. Decellularization was performed with perfusion through the brachial artery of 1% SDS (30 days) and 1% Triton X (15 days). Tissue biopsies confirmed the effective removal of intracellular components from all the tissues (i.e., muscle, nerve, skin, vessels, tendon, cartilage, and bone). Recellularization was not tried in this study.

Face allotransplantation has recently been developed for severe disfigurement [354] but concerns remain for the need of lifelong immunosuppression and chronic rejections, which may occur even after many years. The possibility to develop engineered face grafts involving human ECM recellularized and autologous cells would represent a valid alternative. With this aim, decellularization of partial and total human face grafts have recently been performed, together with trials of recellularization [355]. In particular, decellularization was performed with perfusion through the arterial facial arteries of 1% SDS and 1% Triton X 100 (first sequence), 2-propanol (second sequence), and DNase I (third sequence). In order to evaluate the suitability of the grafts for transplantation, the total decellularized face was implanted on the original donor head, with effective integration and congruence at CT. Moreover, a lower face graft was reperfused for 4 h in a porcine recipient through anastomosis of the facial arteries to the abdominal aorta and inferior mesenteric artery, demonstrating the functional preservation of the innate vasculature of the scaffold. In this study, recellularization procedures were also performed. In particular, dermal fibroblasts and mouse myoblast progenitors were seeded in discs of acellular lips, showing viability, attachment and proliferation but limited deep migration. Whole lips were also recellularized through bioreactor perfusion with human aortic endothelial cells and mouse myoblast progenitors; typical endothelial cells were then visible on the luminal surface of both large and small vessels and viable myoblastic cells were also found in groups at different depth.

## 27. Conclusions and Future Perspectives

In the present paper, we reviewed literature about tissue engineering approaches involving decellularization of human tissues and organs. In the most recent years, more and more attention has been put on human sources, due to increased awareness about the importance of the specificity of extracellular matrix, in terms of architectural, biomechanical, and bioactivity characteristics. A wide and increasing series of human structures have been considered but some other tissues/organs could be of interest, such as seminal vesicles, spleen, and ductal, fascial, or meningeal structures. It is also important to stress that ECM derived from decellularization of a certain tissue may be used for tissue engineering of another tissue type. An engineered organ, in fact, may benefit from an ECM with different biomechanical characteristics from native tissue; for instance, with reference to techniques of surgical implantation. To this purpose, technological advancements may address this need. The advent of 3D bioprints and computer-aided design (CAD) technologies would be helpful in manufacturing tissue substitutes that, combining the biological properties of the ECMs with tunable mechanical and morphological characteristic, will allow for a progressive customization of therapy [356,357,358,359]. According to our knowledge, few attempts are reported in literature about the development of human-derived bioinks [360,361]; however, they may be considered as a milestone in this research field. 

Human tissues may be obtained from surgical or cadaveric materials. As confirmed for face [355] and venous segments with valves [362], tissues sampled from cadavers can be used to obtain grafts through decellularization even several days after death, due to resistence of ECM to degradation. Moreover, routinely performed washing and sterilization procedures assure for safe implantable scaffolds, free from any residual chemical/biological reagent or contaminants which may compromise the in vivo outcome of the implant. In this sense, we have previously stressed the potential role that Body Donation Programs could play as Human Tissue BioBanks [12]. 

Trials of recellularization have been performed with most human grafts but many further approaches will have to be considered. The most promising cell types include mesenchymal stem cells and induced pluripotent stem cells, due to their availability and capability of differentiation along different lineages, also on the basis of stimuli from the extracellular matrix. In the future, it will be particularly interesting to evaluate the suitability of these cells also for repopulation of decellularized skeletal muscle or other parenchymal organs, such as kidney and pancreas. Moreover, some types of stem cells have not yet been consistently studied for recellularization purposes; only few reports, for instance, considered the potentialities of human amniotic mesenchymal stem cells.

Future developments of in vitro recellularization models may be achieved by the use of innovative and dedicated bioreactors to better recreate the physiological microenvironment for cultures in comparison to conventional static conditions [363]. Dynamic cell culture on support matrices assures for a constant flow of fresh nutrients and respiratory gases which enhances cell viability, proliferation, and differentiation, resulting in the development of a more uniform and functional tissue. Moreover, bioreactor systems can reduce processing time and handling steps, limiting contamination risk. Even more interestingly, fluid flow-based in vitro culture exposes cells to the mechanical stimulation of shear stress, which may represent a key point for the engineering of several tissues (e.g., vessels, heart valves, and bone) [364].

Although excellent results have been reported in literature about decellularization and in vitro recellularization of various human tissues/organs, a lot of work is still to be made about approaches of surgical implantation. Apart from tests of immunogenicity, successful orthotopic implantations of functional human grafts in animals included few structures (cornea and cartilage). Particularly intriguing are the efforts to develop human grafts of very complex organs (liver, kidney, or lung) as their functional complexity obviously derives from tissue architectures, which are quite difficult to achieve in in vitro repopulation 

Ameliorating in vitro engineering of extracellular matrices and promoting research and development of advanced devices will significantly contribute to customization of therapies. To date, existing limitations about biodevices are related to the novelty of this field and concern the fact that tissue-engineered products are mainly developed by enterprises of small and medium sizes, universities, and academies, for which regulatory experiences are limited. Conversely, proving the safety of such products is required and only the existence of licenses allows for the preclinical evaluation of human-derived bioengineered products. 

Proving the in vivo efficacy of tissue-engineered products holds great promise for the development of “off the shelf materials” to be promptly used in case of tissue damage or injury.

## Figures and Tables

**Table 1:** Clinical trials. Human implantation of decellularized extracellular matrix (ECM) allografts.

Implanted Tissue	Pathology	Type of Scaffolds	Recipient(s)	Follow-up	Findings/Complications	Reference
Trachea	- End-stage bronchomalacia	Decellularized trachea by detergent enzymatic method + autologous epithelial cells and mesenchymal stem-cell-derived chondrocytes	n = 1 (age 30 years)	4, 14 days; 2, 4 months	- Uneventful postoperative course - functional and normal airways - no anti-donor antibodies/immunosuppressive therapy	Macchiarini et al., 2008 [28]
				5 years Approximately every 3 months: multidetector CT scan and bronchoscopy; every 6 months mucosal biopsy samples	- Cicatricial stenosis close to anastomosis by 12 months; repeated endoluminal stenting was required - the tissue-engineered trachea remained open, well vascularised, completely recellularized with functional respiratory epithelium - no stem-cell-related teratoma neither anti-donor antibodies - normal lung function/cough reflex and social/working life	Gonfiotti et al., 2014 [37]
	Long-segment congenital tracheal stenosis and pulmonary sling	Decellularized trachea by detergent enzymatic method + bone marrow mesenchymal stem cells + patches of autologous epithelium	n = 1 (age 10 years)	2 years	- Functional airways - revascularization - initial strong local neutrophil response - not evident cytological evidence of epithelium restoration for 1 year - no focal biomechanical strength - normal chest CT scan and ventilation-perfusion scan - grown of 11 cm in height - the patient returned to school	Elliott et al., 2012 [30]
				4 years	- Restriction of growth within an area of in-stent stenosis - calculation of tracheal airflow - differentiated respiratory layer with complete mucosal lining at 15 months, despite retention of a stent - no abnormal immune activity - increased velocity and pressure drops around a distal tracheal narrowing	Hamilton et al., 2015 [31]
Heart valves	- Aortic or pulmonary dysfunctions	Decellularized pulmonary valves for Ross aortic valve or dysfunctional pulmonary allograft replacements	n = 36 (age 0.27–51.2 years)	1 and 3 months	- No panel reactive antibody response, except for 1 patient who had a prior xenograft aortic valve replacement - normal allograft valve function	Elkins et al., 2001 [57]
	- Aortic stenosis - aortic insufficiency	Decellularized pulmonary allografts for right ventricular outflow tract reconstruction during the Ross procedure	n = 11, decellularized graft (mean age 23.0 ± 9.04 years) n = 9, cryopreserved graft (mean age 24.3 ± 8.06 years)	5, 10 days; 1, 3, 6, 12 months	- Lower panel reactive antibody levels in decellularized valves than the cryopreserved ones - mild gradients increase only in cryopreserved valves - no cases of pulmonary insufficiency	da Costa et al., 2005 [58]
	- Congenital oracquired aortic valve disease, - aortic aneurysm - aortic valve endocarditis	Decellularized aortic valve conduit for aortic root replacement	n = 22 (mean age 53 ± 14 years)	1, 3 months; 1 year	- Low panel reactive antibody response against the homograft - low transvalvular gradients - no early mortality - Two late deaths at 1 postoperative year, unrelated to the homograft - postoperative blood transfusion for 12 patients	Zehr et al., 2005 [59]
	Congenital pulmonary valve failure	Pulmonary heart valves engineered with autologous endothelial progenitor cells	2 pediatric patients (age 11 and 13 years)	3, 6, 12, 18, 24, 30, 36, and 42 months	- Increase of the valvular annulus diameter - decrease of valve regurgitation - decrease or increase of mean transvalvular gradient - unchanged or decreased right-ventricular end-diastolic diameter - increase of the body surface area - the grafts remodelled and grew with the somatic growth of the patients - no signs of pulmonary dilatation or stenosis, valve degeneration, cusp thickness, or reduction of cusp’s mobility	Cebotari et al., 2006 [60]
	- Aortic stenosis - aortic insufficiency	Decellularized pulmonary allografts in patients undergoing a Ross procedure	n = 68 (mean age 30.3 ± 11.2 years)	4 years	- Increase of transvalvular gradients - partial reendothelization and progressive and progressive repopulation of the tunica media with autogenous cells - 1 early death and 1 late death - 2 reoperations for endocarditis - no progressive pulmonary insufficiency	Costa et al., 2007 [61]
	Isolated aortic valve disease or multilevel left ventricular outflow tract obstruction	Decellularized pulmonary conduit implanted during Ross operation with right ventricular outflow tract reconstruction	n = 183 (mean age 23.3 ± 15.2 years): n = 156, cryopreserved homograft n = 22, decellularized homograft n = 5, bovine jugular vein	5.7 ± 3.3 years (mean follow-up)	- Peak systolic right ventricular outflow tract gradient exceeding 20/40 mm Hg - right ventricular outflow tract insufficiency (> 2+ regurgitation) - Two early deaths and one late death - Eight reoperated patients for significant obstruction of the pulmonary homograft	Brown et al., 2008 [62]
	- Aortic stenosis - aortic insufficiency - mixed lesion	Decellularized aortic valve conduit for aortic root replacement	n = 41 (mean age 34 years)	19 months	- Decreased transvalvular gradients - none or mild valvular regurgitation - stable root dimensions - discrete conduit calcification - Three early deaths and 1 late death - One reoperation due to a failed mitral valve repair - no reoperation for valvular graft failure	da Costa et al., 2010 [63]
	- Aortic stenosis - aortic insufficiency	Decellularized pulmonary allografts in patients undergoing a Ross procedure	n = 63 (mean age 28.6 ± 16.0 years): n = 29, decellularized allograft n = 34, standard cryopreserved allograft	4.9 ± 2.7 years (mean follow-up)	- The median peak gradient at discharge was 12 mm Hg and was not significant at last follow-up - no deterioration in conduit valve function in the decellularized allograft group - mild/moderate conduit regurgitation in several patients of the cryopreserved allograft group - no early or late deaths or significant morbid events - no conduit dysfunction nor reoperations	Brown et al., 2011 [64]
	- Pulmonary atresia - pulmonarystenosis and/or insufficiency	Decellularized pulmonary homografts	n = 38, decellularized homograft (mean age 16.4 ± 11.4 years) n = 38, cryopreserved homograft (mean age 16.6 ± 11.3 years) n = 38, bovine jugular vein (mean age 17.9 ± 12.5 years)	5 years	- Lower transvalvular gradient and improved freedom from explantation in decellularized homograft patients - no increase of cusp thickening or aneurysmatic dilatation in decellularized homograft vs cryopreserved/bovine homograft patients - moderate regurgitation in three decellularized homograft patients - 1 late death, not related to valve allograft implantation	Cebotari et al., 2011 [65]
	- Aortic stenosis - aortic insufficiency - aortic aneurysm - double aortic lesion	Cellularized and decellularized aortic/pulmonary allografts for aortic valve replacement	n = 6, cellularized allograft (median age 59 years) n = 6, decellularized allograft (median age 38.6 years)	5, 10 days; 1, 3 months	Decellularized grafts elicited lower levels of anti-HLA class I and II antibody formation after implantation than cellularized allografts	Kneib et al., 2012 [66]
	Congenital pulmonary valve malformations	Decellularized pulmonary homografts for pulmonary valve replacement	n = 93, decellularized homograft (mean age 15.8 ± 10.21 years) n = 93, cryopreserved homograft (mean age 15.9 ± 10.4 years) n = 93, bovine jugular vein conduit (mean age 15.6 ± 9.9 years)	10 years	Reduced reoperation rates for decellularized pulmonary homografts in comparison with cryopreserved and bovine homograft	Sarikouch et al., 2016 [67]
	- Pulmonary stenosis - pulmonary insufficiency	Decellularized pulmonary allografts for pulmonary valve replacement	n = 163, decellularized allograft (mean age 207.6 ± 197.8 months) n = 124, standard allograft (mean age 151.5 ± 171.5 months)	60.1 ± 37.1 months (mean follow-up)	- Lower conduit dysfunction and pulmonary insufficiency and stenosis among patients receiving decellularized allografts - Thirteen early deaths and 15 late deaths, not attributable to conduit structural failure - significant conduit dysfunction in 76 of the survived patients	Bibevski et al., 2017 [68]
	- Pulmonary stenosis - pulmonary insufficiency	Decellularized pulmonary valves	n = 5 adult patients n = 1 pediatric patients	3, 6 months; 1, 2, 3 years	- The valve and ventricular function were significantly improved and maintained postoperatively - no complications related to decellularized heart valve implantation were observed	Ozawa et al., 2018 [69]
Vessels-pulmonary artery	Right ventricular outflow tract reconstruction in neonates and infants	MatrACELL (LifeNet Health, Inc.) supplied in three forms: - thin patches - thick patches - hemipulmonary arteries	n = 44 (mean age 290 ± 343 days) n = 26, thin patches n = 1, 2 thin patches n = 10, thick patches n = 1, thick and thin patch n = 9, hemipulmonary artery	230.3 days	- Four severe disease-related complications, unrelated to the patch repair - 41 no device-related adverse events - One elective surgical removal of the thin allograft	Lofland et al., 2011 [70]
	Congenital cardiac reconstructions	MatrACELL	n = 108 (mean age 367 ± 655 days)	687 patient-days	- Two were lost and excluded from analyses. - 106 had no device-related serious adverse events or failures	Hopkins et al., 2014 [71]
Vessels-iliac or mammary artery	Hepatic portal vein thrombosis	Human iliac or mammary veins from deceased organ donors Decellularization by 1% Triton X-100 and 1% tri-*n*-butyl phosphate and 4 mg/L DNAse. Recellularization by autologous blood-perfusion.	n = 2 (4 years and 2 years)	1 year and 9 months 1 year and 7 months	- Patient 1: portal pressure from 22 cm to 14 cm after reperfusion of the vein; 30–40 cm/s flow velocity both in the portal vein and hepatic artery; diminishing of the esophageal varices; normal clinical laboratory parameters and no surgical/other complications. - Patient 2: a revision occurred at day 2 due to a narrowing at the site of anastomosis in the liver; uneventful postoperative period with good intrahepatic blood flows. Reduced diameter at both anastomotic sites at month 6 with thrombosis of the graft. Revision of the graft at month 7, the patient received a second vein but poor perfusion of the right portal system occurred the day after; a good perfusion was observed at day 4.	Olausson et al., 2014 [72]
Cadaver vein	Patients with renal failure without adequate vasculature for creation of a native arteriovenous fistula	Placement of an synergraft-processed cadaver vein allograft for hemodialysis access.	n = 20	1 year	- None of the synergraft patients became allosensitized at 3 months after engraftment; - no allograft was lost to infection.	Madden et al., 2002 [73]
Bladder	Hypospadias	Decellularized bladder submucosal, collagen matrix for urethral reconstruction	n = 4 (4–20 years)	22 months	- Satisfactory cosmetic and functional results - development of a subglanular fistula in 1 patient who had a 15 cm neourethra reconstruction - no episodes of infection	Atala et al., 1999 [74]
	Urethral strictures	Decellularized bladder submucosal collagen matrix for urethral reconstruction	n = 28 (mean age 40.4 years)	36, 48 months	- Formation of a wide patent urethra in 24 of the 28 patients - increase of the mean maximum urine flow rate - adequate caliber conduits; normal urethral tissue appearance - formation of the typical urethral stratified epithelium - slight caliber decrease at the anastomotic sites in four patients - development of a subcoronal fistula in one patient which closed spontaneously within 1 year	El-Kassaby et al., 2003 [75]
	Myelomeningocele resulting in a poorly compliant bladder	Decellularized bladder matrix seeded with autologous urothelial and muscle cells	n = 7 (4–19 years)	22, 61 months	- Increase of bladder volume and compliance - Obtainment of an adequate bladder structural architecture and phenotype - normal mucus production and preserved renal function - no metabolic consequences nor urinary calculi formation	Atala et al., 2006 [76]
	Urethral strictures	Decellularized bladder submucosal, collagen matrix for urethral reconstruction	n = 30 (mean age 36.2 years): n = 15, buccal mucosa-derived graft n = 15, bladder-derived graft	18, 6 months	- Significant voiding improvement - formation of normal urethral tissue - bladder matrix grafts assured the best outcome (i.e., comparable to the buccal mucosa grafts) in patients with a healthy urethral bed - no spongiofibrosis and good urethral mucosa	El-Kassaby et al., 2008 [77]
Amniotic membrane	Chronic nonhealing recalcitrant diabetic partial- or full-thickness foot ulcers.	Biovance (Celgene Cellular Therapeutics)	n = 13 patients (+1) * (mean age 63.4 years) * 1 patient received Biovance on bilateral feet on separate occasions so that was considered as 2 separate subjects.	12 weeks	- Nine completed the study without deviation/adverse reactions n = 5, 100% wound closure; n = 3, significant decrease in wound size (i.e., 50 to 80%); n = 1, <50% wound clousure	Letendre et al., 2009 [78]
Cornea	Keratoconus	Decellularized corneal stromal lamina seeded with autologous adipose-derived adult stem cells	n = 9 (mean age: 34 years): n = 5, acellular graft n = 4, recellularized graft	1 day; 1 week; 1, 3, 6 months	General improvement of: - all visual parameters - refractive sphere and anterior keratometric values - spherical aberration and total higher order aberrations - no clinical haze or scarring	Alió del Barrio et al., 2018 [79]

CT, computed tomograph.

**Table 2:** Skeletal muscle and tendons. Decellularization techniques, biomechanical tests, recellularization methods, and in vivo implant of human muscular and tendinous extracellular matrix.

Tissues	Decellularization Methods	Biomechanical Tests	In Vitro Recellularization	In vivo Implant	References
Tibialis anterior and abdominal rectus muscle	4% SDS + DNase I	Uniaxial tensile tests	-	Rabbits: surgical defect of the abdominal wall	Porzionato et al., 2015 [14]
	0.05% trypsin and 0.02% EDTA + 2% Triton X-100 and 0.8% NH_4_OH (*)				
Rectus femoris and supraspinatus muscles	1% SDS with 1 % EDTA in Tris–HCl + DNase/RNase buffer (1 kU/mL)	Uniaxial tensile tests	-	Outbred mice: dorsal subcutaneous pouch	Wilson et al., 2016 [86]
Hemidiaphgram	Freezing at −80 °C	-	-	Dog: native diaphgram replacement	Davari et al., 2016 [87]
	dH_2_O + 4% sodium deoxycholate and 4000 KU of DNase-1 in 1 mol/L NaCl (*)				
Upper limb flexor tendons	0.1% EDTA + 1% Triton X-100 and 0.1% EDTA	Uniaxial tensile tests	Seeding of human dermal fibroblasts	-	Pridgen et al., 2011 [88]
	0.1% EDTA + 1% Tri-n-butyl-phosphate and 0.1% EDTA				
	0.1% EDTA + 1% SDS and 0.1% EDTA				
	0.1% EDTA + 0.1% SDS and 0.1% EDTA (*)				
Upper limb flexor tendons	0.1% EDTA + 0.1% SDS and 0.1% EDTA (2%, 5%, and 10% peracetic acid solutions)	Uniaxial tensile tests	Seeding of human dermal fibroblasts	-	Woon et al., 2011 [89]
Upper limb flexor tendons	0.1% EDTA + 0.1% SDS	Uniaxial tensile tests	-	Rats: dorsal subcutaneous tissue anchored to spinal ligaments (immunogenicity test)	Raghavan et al., 2012 [90]
Upper limb flexor tendons	0.1% EDTA + 0.1% SDS and 0.1% EDTA	Uniaxial tensile tests on unseeded and reseeded tendons after 2 or 4 weeks from surgical implantation	Seeding of adipose-derived mesenchymal stem cells	Athymic rats: unseeded and reseeded sutured tendons in dorsal subcutaneous tissue	Schmitt et al., 2013 [91]
Upper limb flexor tendons	0.1% EDTA + 0.1% SDS + DNase and 1M NaCl	-	Injection of adipose-derived mesenchymal stem cells	-	Martinello et al., 2014 [92]
Upper limb flexor tendons	0.1% EDTA + 0.1% SDS and 0.1% EDTA	-	Seeding or injection with FBS or hydrogel of adipose-derived mesenchymal stem cells	-	Long et al., 2017 [93]
Upper limb flexor tendons from cadavers	Trypsin and 0.05% EDTA	-	Seeding on coated sutures of bone marrow-derived mesenchymal stem cells	-	Le et al., 2018 [94]
Flexor digitorum profundus tendon with attached distal phalanx	5% peracetic acid + 0.1% EDTA + 0.1% SDS	Uniaxial tensile tests	-	-	Bronstein et al., 2013 [97] Fox et al., 2013 [98]
	ultrasonication (3 min)				
	ultrasonication (10 min)				
	ultrasonication (10 min) + 5% peracetic acid + 0.1% EDTA + 0.1% SDS (*)				
Achilles tendon	hypotonic aqueous solutions + trypsin digestion + peracetic acid + Triton X-100	Tensile testing	Seeding of NIH 3T3	Rabbits: anterior cruciate ligament reconstruction	Whitlock et al., 2012 [95]
Flexor digitorum profundus, superficialis, and pollicis longus tendons from fresh-frozen, human cadaveric hands and upper extremities	0.1% EDTA + 0.1% EDTA and 0.1% SDS + lyophilized + milled + pepsin/hydrochloric acid + gelation	-	-	Rats: full-thickness defect within the mid-substance of each Achilles tendon	Chiou et al., 2015 [96]

SDS, sodium dodecyl sulfate; DNase, deoxyribonuclease; + means that separate cycles were performed; ‘and’ means that a mixture was performed between different substances; (*) best protocol.

**Table 3:** Adipose tissues. Decellularization techniques, biomechanical tests, recellularization methods, and in vivo implant of adipose extracellular matrix.

Tissues	Decellularization Methods	Biomechanical Tests	In Vitro Recellularization	In Vivo Implant	References
Subcutaneous adipose tissue from abdomen, breast, omentum, pericardial depot, thymic remnants	Freezing–thawing cycles + 0.25% trypsin and 0.1% EDTA + 99.9 2-propanol + 8 g/L NaCl, 200 mg/L KCl, 1 g/L Na_2_HPO_4_, 200 mg/L KH_2_PO_4_ + 55 mM Na_2_HPO_4_, 17 mM KH_2_PO_4_, 4.9 mM MgSO_4_, DNase, RNase, lipase	Indentation and compression tests	Static/dynamic seeding on ECM fragments/microcarriers/bead foams or encapsulation into hydrogel scaffolds of: - human adipose-derived stem cells - rat adipose-derived stem cells - human preadipocytes - chronic wound human fibroblasts	Rats and athymic mice: subcutaneous implantation	Flynn, 2010 [104] Turner and Flynn, 2012 [107] Turner et al., 2012 [108] Porzionato et al., 2013 [13] Yu et al., 2013 [112] Omidi et al., 2014 [102] Cheung et al., 2014 [113] Han et al., 2015 [114] Brown et al., 2015 [115] Baker et al., 2017 [103] Yu et al., 2017 [116] Shridhar et al., 2017 [117] Morissette Martin et al., 2018 [119]
	1% SDS + 2.5 mM sodium deoxycholate, lipase, and colipase	-	Seeding on hydrogel scaffolds of human adipose-derived stem cells	Athymic mice: injection of acellular adipose matrix in the subcutaneous dorsal region	Young et al., 2011 [106]
	2.5 mM sodium deoxycholate + lipase and colipase				
	Mechanical agitation + 0.1, 1, 3, 5% peracetic acid + 1% Triton X-100 in 2 mM EDTA + 600 U/mL DNase	-	Seeding on hydrogel ECM of human adipose-derived stem cells	Rats: subcutaneous injection	Wu et al., 2012 [109]
Adipose tissue surgically sampled from abdomen, breast and forearm	Ultrasonic homogenization + 0.25% pancreatin	-	Culture of human adipose-derived stem cells with ECM microparticles	Nude mice: subcutaneous implantation of cell/scaffold complexes	Wang et al., 2013a [110]
	Freezing–thawing cycles + 0.5-1 M NaCl + 0.25% trypsin/EDTA + 1% Triton X-100	-	Seeding on adipose tissue microparticles of human adipose-derived stem cells	Nude rats: subcutaneous injection	Wang et al., 2013b [111]
	Decellularization according to Flynn, 2010 [104]	-	-	-	Sano et al., 2014 [126]
	Freezing–thawing cycles				
	DNase + 4% SDS + 1% sodium azide				
	1% Triton X-100 + DNase				
	Freezing–thawing cycles + 0.25% trypsin/EDTA + 1% Triton X-100 + isopropanol	-	Seeding of breast cancer cells	-	Dunne et al., 2014 [127]
	1% SDS + 2.5 mM sodium deoxycholate + 100 µg/ml lipase and 50 ng/ml colipase	Compression tests	Encapsulation into the composite hydrogels of rat adipose-derived stem cells	Rats: subcutaneous tissue	Kayabolen et al., 2017 [118]
	Freezing–thawing cycles + 0.5% trypsin + 99.9% isopropanol + 500 U/mL Benzonase^®^	-	-	Mice: subcutaneous dorsal tissue	He et al., 2018 [120]
	Decellularization according to Flynn, 2010 [104]	-	-	GFP^+^ transgenic mice: subcutaneous tissue	Thomas-Porch et al., 2018 [121]
	1 M NaCl + 1 mM EDTA + Lysis buffer (1% tergitol type NP-40, 0.1% SDS, 5 mM EDTA, 0.4M NaCl, 50 mM Tris-HCl pH 8, 1 mM PMSF)				
	Homogenization + 2 M urea buffer				
Subcutaneous adipose tissue from cadavers	Organic solvent + surfactant/ethanol based solution	-	-	Immunocompetent and athimic mice: subcutaneous tissue	Giatsidis et al., 2018 [125]
Adipose tissue from cosmetic, abdominoplasty and liposuction surgery	Homogenization + freezing in 2 M urea buffer + 0.5% pepsin in 0.5 M acetic acid	Compression tests	Encapsulation into ECM-PEG hydrogels of human adipose-derived stem cells	Mice: subcutaneous implant of non-seeded ECM-PEG hydrogels	Li et al., 2018 [122]
	Freezing–thawing cycles + 99.9% isopropanol 0.05% trypsin/0.05% EDTA, 20 ng/mL DNase and 20 ng/ml RNase	Tensile tests	Seeding of human adipose-derived stem cells	-	Song et al., 2018 [123]
	Freezing–thawing cycles + homogenization + 1% Triton X-100 + 100 U/mL DNase and 100 µg/ml RNase	-	Encapsulation into ECM hydrogels of human adipose-derived stem cells	Rats: subcutaneous injection of unseeded hydrogel scaffolds	Zhao et al., 2018 [124]

SDS, sodium dodecyl sulfate; PEG, poly(ethylene glycol); DNase, deoxyribonuclease; RNase, ribonuclease; PMSF, phenylmethylsulfonyl fluoride; + means that separate cycles were performed; ‘and’ means that a mixture was performed between different substances.

**Table 4:** Cardiac tissues. Decellularization techniques, biomechanical tests, recellularization methods, and in vivo implants of human heart extracellular matrix.

Tissues	Decellularization Methods	Biomechanical Tests	In Vitro Recellularization	In Vivo Implants	References
Myocardium	10 µM Tris buffer and 0.1% EDTA + 0.5% SDS + 50 U/mL DNase and 1 U/mL RNase	Uniaxial tensile test	Seeding on composite scaffolds (acellular myocardium/fibrin hydrogel) of mesenchymal progenitor cells	Nude rat model of acute and chronic cardiac infarction: acellular and recellularized composite scaffolds as patches on the infarcted myocardium	Godier-Furnémont et al., 2011 [145]
Myocardium (300 µm thick-sections)	0.5% SDS	-	Seeding of: - cord blood-derived mesenchymal stemcells; - murine cardiomyocytes derived from induced pluripotent stem cells; - murine neonatal cardiomyocytes	-	Oberwallner et al., 2014 [138]
	5% Triton X-100				
	4% sodium deoxycholate				
	10 mM Tris and 0.1% EDTA + 0.5% SDS + FBS (*)				
Myocardium	1% deoxycholic acid + DNase and RNase	-	Injection of cord blood mononuclear cells on nanofibers-coated myocardial ECM	Sheep models of myocardial infarction: ischemic myocardial apex	Guhathakurta et al., 2014 [142]
Heart (whole organ)	Perfusion with: 1% SDS	Pressure–volume measurements	Seeding on slices of decellularized human leftventricle of: - human cardiac-progenitor cells - bone-marrow mesenchymal stem cells - human umbilical vein endothelial cells - H9c1and HL-1 cardiomyocytes	-	Sánchez et al., 2015 [137] Sánchez et al., 2016 [139]
Heart (whole organ)	Decellularization according to Sanchez et al., 2015 [97]	-	Seeding on myocardial left ventricle slices of cardiac-like cells derived from induced pluripotent stem cells	-	Garreta et al., 2016 [140]
Heart (whole organ)	Perfusion with: 1% SDS + dH_2_O + 1% Triton X-100 + 25 U/mL DNase	Biaxial tensile test	Perfusion of whole acellular heart / seeding of decellularized cardiac slices with cardiac myocytes derived from human induced pluripotent stem cells	Rats: decellularized myocardium fragments into subcutaneous tissue	Guyette et al., 2016 [141]
Myocardium	1% SDS + DNase and RNase	-	-	-	Johnson et al., 2016 [146]
Myocardium (300 μm thick-slices)	10 mM Tris and 0.1% EDTA + 0.5% SDS + FBS	-	Murine HL-1 cardiomyocyte cultures	-	Kappler et al., 2016 [143] Becker et al., 2017 [144]
Myocardium (350 μm thick-sections)	10 mM Tris and 0.1% EDTA + 0.5% SDS + FBS	-	Seeding of human cardiac progenitor cells	-	Di Meglio et al., 2017 [148]
	10 µM Tris and 0.1% EDTA + 0.5% SDS + 50 U/mL DNase and 1 U/mL RNase				
	10 mM Tris-HCl and protease inhibitors + 0.1% SDS + 50 U/mL DNase and 1 U/mL RNase				
	1% SDS + dH_2_O + 1% Triton X-100				
	1% SDS and L1% Triton X-100 (*)				
Myocardium	Decellularization according to Johnson et al., 2016 [103]	-	-	Humanized mice: injection of acellular ECM hydrogels in the subcutaneous dorsal tissue	Wang et al., 2017 [147]
Myocardium	Decellularization according to Becker et al., 2017 [96]	Uniaxial pulling tests	Seeding on myocardial ECM hydrogel/amniotic membrane composite scaffolds of: - human cardiac fibroblasts; - human epicardial progenitor cells; - murine HL-1 cardiomyocytes	-	Becker et al., 2018 [144]
Pericardium	10 mM Tris-HCl and protease inhibitors + 0.1% SDS + 50 U/mL DNase and 1 U/mL RNase	Uniaxial tensile test	-	-	Mirsadraee et al., 2006 [150]
	Decellularization according to Mirsadraee et al., 2006 [106]	-	Seeding of human dermal fibroblasts from human skin of cadaveric donors	Mice: acellular pericardia into the subcutaneous tissue	Mirsadraee et al., 2007 [149]
	Decellularization according to Mirsadraee et al., 2006 [106]	Uniaxial tensile test	-	Immune-competent mice: subcutis	Vinci et al., 2013 [151]
	Hypotonic/hypertonic solutions + 1% SDS	-	-	Rats: injectable matrix gels into the left ventricular wall	Seif-Naraghi et al., 2011 [156]
	0.1% SDS and protease inhibitors + DNase/RNase	Uniaxial compression and tensile tests	Seeding on gel scaffolds of human cardiac progenitor cells	Rats: cell-loaded scaffolds into the subcutaneous tissue	Rajabi-Zeleti et al., 2014 [152]
	0.1% SDS + Triton X-100 + 0.1 mg/mL DNase	Tensile tests	Seeding on ECM/hydrogel RAD16-I scaffolds of porcine mediastinal adipose-derived progenitor cells	Porcine models of myocardial infarction: repopulated scaffolds on the ischemic myocardium	Prat-Vidal et al., 2014 [157] Gálvez-Montón et al., 2017 [158] Perea-Gil et al., 2018 [159]
	Acetone + Ethanol + 1 N NaOH + 7% NaCl + H_2_O_2_	Uniaxial mechanical resistance tests	-	Rats: decellularized human pericardium patches on the abdominal aorta	van Steenberghe et al., 2017 [153]
	Decellularization according to van Steenberghe et al., 2017 [109]	Uniaxial mechanical resistance tests	-	In vivo implant according to van Steenberghe et al., 2017 [153]	van Steenberghe et al., 2018 [154]
	Decellularization according to van Steenberghe et al., 2017 [109]	-	-	Vietnamese pigs: decellularized ECM fragments/patches into subcutaneous dorsal tissue or on carotid/aorta	van Steenberghe et al., 2018 [155]

SDS, sodium dodecyl sulfate; DNase, deoxyribonuclease; RNase, ribonuclease; dH_2_O, deionized water; + means that separate cycles were performed; ‘and’ means that a mixture was performed between different substances; (*) best protocol.

**Table 5:** Heart valves. Decellularization techniques, biomechanical tests, recellularization methods, and in vivo implants of human heart valve extracellular matrix.

Tissues	Decellularization Methods	Biomechanical Tests	In Vitro Recellularization	In Vivo Implants	References
Aortic and pulmonary valves/conduits	SynerGraft treatment: dH_2_O + DNase and RNase	Uniaxial tensile test	-	Patients with heart valve disfunctions	Elkins et al., 2001 [57] Zehr et al., 2005 [59] Brown et al., 2008 [62] Brown et al., 2011 [64] Bibevski et al., 2017 [68]
Aortic and pulmonary valves	0.5% Trypsin and 0.2% EDTA	-	Dynamic seeding of human endothelial cells from saphenous vein	-	Cebotari et al., 2002 [168] Cebotari et al., 2006 [65]
Pulmonary valve conduits	0.05% Triton 100-X, 0.05% sodium deoxycholate and 0.05% octylphenyl-polyethylene glycol + 150 IU/DNase and 100 µg/ml RNase	-	-	-	Rieder et al., 2005 [166]
	1% deoxycholic acid + 70/80% ethanol	-	-	Patients with heart valve disfunctions	da Costa et al., 2005 [58] Costa et al., 2007 [61]
	0.1% SDS	.	.	Patients with heart valve disfunctions	Costa et al., 2007 [61] da Costa et al., 2010 [63] Kneib et al., 2012 [66]
Pulmonary valves	Hypo/hypertonic solutions 0.1–1% Triton X-100 + 10 mM Sodium cholate + Benzonase^®^	-	Seeding of human bone marrow mesenchymal stem cells	-	Iop et al., 2009 [167]
	0.5% sodium deoxycholate and 0.5% SDS + 0.9% NaCl	-	-	Patients with heart valve disfunctions	Cebotari et al., 2011 [65] Sarikouch et al., 2016 [67] Ozawa et al., 2018 [69]
Aortic valves	0.5% Trypsin + 20 mg/mL RNase	-	Seeding of cardiac mesenchymal stromal cells from human auricle fragments	-	Dainese et al., 2012 [162]
Aortic and pulmonary valves	SynerGraft treatment: dH_2_O + DNase and RNase	-	-	-	Gerson et al., 2012 [169]
	Hypo/hypertonic solutions + 0.05% Triton 100-X + Benzonase^®^	Torsional wave experiments	-	-	Jiao et al., 2012 [170]
	1% SDS and 0.05% NaN_3_	Tensile strength measurement	Dynamic perfusion of human umbilical vein endothelial cells on the luminal surface	-	Weymann et al., 2013 [171]
Biohybrid aortic valves	0.5% sodium deoxycholate and 0.5% SDS	-	Dynamic seeding of fibroblasts and endothelial cells from human saphenous vein	-	Koening et al., 2016 [163]
Aortic valves Aortic valves	Hypertonic salt solution + 0.05% Triton X-100 + Benzonase^®^	Biaxial mechanical testing	-	-	VeDepo et al., 2017 [164]
	Hypo/hypertonic solutions 0.1–1% Triton X-100 + 10 mM Sodium cholate + Benzonase^®^		Seeding of human bone marrow mesenchymal stem cells		Iop et al., 2017 [165]
Aortic and pulmonary valves	10 mM Tris, 0.1% EDTA, 10 KIU/L aprotinin + 0.1% SDS + Nuclease treatment	Uniaxial tensile test	-	Mouse: subcutis	Vafaee et al., 2018 [172]
Mitral valves	0.5–1% SDS	-	Seeding of post-infarct murine bone marrow c-kit^+^ cells	Murine models of myocardial infarction: repopulated scaffolds on the infarcted epicardium/myocardium	Wan et al., 2017 [178]

dH_2_O, deionized water; SDS, sodium dodecyl sulfate; DNase, deoxyribonuclease; RNase, ribonuclease; + means that separate cycles were performed; ‘and’ means that a mixture was performed between different substances.

**Table 6:** Buccal cavity. Decellularization techniques, biomechanical tests, and recellularization methods of human buccal cavity extracellular matrices.

Tissues	Decellularization Methods	Biomechanical Tests	In Vitro Recellularization	References
Gingiva	Liquid nitrogen + 1% SDS + 1% Triton X-100	-	Seeding of rat bone marrow mesenchymal stem cells	Mahdavishahri et al., 2012 [234]
	Liquid nitrogen + 0.1% SDS	-	Seeding of rabbit blastema cells	Naderi et al., 2013 [235]
	Liquid nitrogen + 0.5% SDS			
	Liquid nitrogen + 1% SDS (*)			
Dental pulp	Collagenase and dispase	Stress-strain and Young’s modulus	Seeding of human osteoblast-like cells (MG-63)	Sangkert et al., 2016 [236]
	Collagenase and dispase	-	Seeding of human osteoblast-like cells (MG-63)	Sangkert et al., 2017 [237]
	2% Triton X-100 and 0.1% NH_4_OH	-	Seeding of human stem cells of the apical papilla cell-line	Song et al., 2017 [238]
	0.01 M Tris-HCl and 1 mm EDTA followed by 3 cycles of: 1% SDS + 1% Triton X-100 (*)			
	1% SDS + 1% Triton X-100			
	10 mM Tris and protease inhibitors + [0.1% EDTA and aprotinin (10 KIU mL-1)] and 0.03% SDS and wash in Tris-buffered saline and Tris-hydrochloric acid (50 mM) and DNase (50 U/mL) + RNase (1 U/mL)	-	Seeding of human dental pulp stem cells	Matoug-Elwerfelli M. et al., 2018 [239]
Schneiderian membrane	Liquid nitrogen + 1% SDS	-	Seeding of human adipose tissue mesenchymal stem cells	Rahpeyma et al., 2014 [243]

SDS, Sodium Dodecyl Sulfate; EDTA, ethylene-diamine-tetra-acetic acid; BM-MSCs, bone marrow mesenchymal stem cells; SCAP, human stem cells of the apical papilla; + means that separate cycles were performed; ‘and’ means that a mixture was performed between different substances; (*) best protocol.

**Table 7:** Liver. Decellularization techniques, recellularization methods, and in vivo implant of human hepatic extracellular matrix.

Tissues	Decellularization Methods	In Vitro Recellularization	In Vivo Implant	References
Liver (whole organ or left lobe)	Perfusion with: dH_2_O + 0.025% Trypsin/EDTA + 3% Triton X-100 + 0.01%, 0.1%, 1% SDS	Seeding on cubic scaffolds of: - human hepatic stellate cell line (LX2) - HepG2 cells from hepatocellular carcinoma - Sk-Hep-1 endothelial cells from human adenocarcinoma	Immunocompetent mice: acellular scaffolds into subcutaneous tissue or omentum	Mazza et al., 2015 [249]
Liver (biopsies from HCV-infected patients)	0.5 M NaCl and 10 mM Tris Base + 1% SDS + Mechanical agitation	-	-	Baiocchini et al., 2016 [255]
Liver (whole organ)	Perfusion with: 4% Triton X-100 and 1% NH_4_OH + 0.9% NaCl + DNase solution	Seeding on ECM sections of human umbilical vein endothelial cells	-	Verstegen et al., 2017 [250]
Liver (fragments obtained by a mechanical homogenizer)	1% Triton X-100 and 0.1% SDS + 2% Triton X-100 and 0.1% SDS + 3% Triton X-100 and 0.1% SDS + Mechanical agitation	-	-	Nemets et al., 2017 [254]
Liver (cylindrical tissue samples)	1–0.1% Triton X-100	-	-	Mattei et al., 2017 [251]
	0.1% SDS			
Liver (tissue biopsies sectioned into 50 µm-slices)	0.01% SDS + 0.1% SDS + 0.2% SDS + 0.5% SDS + 1% Triton X-100	Seeding on ECM gel of human induced pluripotent stem cells	-	Jaramillo et al., 2018 [253]
Liver (tissue biopsies sectioned into 50 µm-slices)	dH_2_O + 1% Triton X-100 and 0.1% SDS + 2% Triton X-100 and 0.1% SDS + 3% Triton X-100 and 0.1% SDS + High g-force agitation	Seeding on cubic scaffolds of: - human umbilical vein endothelial cells - human hepatic stellate cell line (LX2) - HepG2 cells from hepatocellular carcinoma Dynamic perfusion of primary human hepatocytes	-	Mazza et al., 2017 [252]

dH_2_O, deionized water; SDS, sodium dodecyl sulfate; DNase, deoxyribonuclease; + means that separate cycles were performed; ‘and’ means that a mixture was performed between different substances.

**Table 8:** Pancreas. Decellularization techniques, biomechanical tests, recellularization methods, and in vivo implant of human pancreatic extracellular matrix.

Tissues	Decellularization Methods	Biomechanical Tests	In Vitro Recellularization	In Vivo Implant	References
Pancreas (whole organ)	Perfusion with: 1% Triton X-100 and 0.1% NH_4_OH	Uniaxial tensile test	Seeding on scaffolds of: - human islet cells - human endothelial cells	-	Peloso et al., 2016 [256]
Pancreas (1 cm^3^ specimens or homogenized tissue)	2.5 mM sodium deoxycholate	-	Seeding on ECM hydrogel-coated plates of: - insulinoma cell line - stem cell-derived beta-like cells - human umbilical vein endothelial cells. Embedding on ECM hydrogel of human embryonic stem cells	Humanized mice: acellular ECM pre-gel into the dorsum	Sackett et al., 2018 [257]

+ means that separate cycles were performed; ‘and’ means that a mixture was performed between different substances.

**Table 9:** Kidney. Decellularization techniques, recellularization methods, and in vivo implant of human renal extracellular matrix.

Tissues	Decellularization Methods	In Vitro Recellularization	In Vivo Implant	References
Kidney (whole organ)	Perfusion with: 0.5% SDS	-	-	Orlando et al., 2013 [260]
	Perfusion with: 1% SDS + 1% Triton X-100	-	-	Song et al., 2013 [262]
	Perfusion with: 0.5% SDS + DNase	-	-	Peloso et al., 2015 [263]
Kidney (cortex samples)	1% SDS	Seeding on ECM gels of: - human kidney peritubular microvascular endothelial cells - human umbilical vein endothelial cells	-	Nagao et al., 2016 [264] Hiraki et al., 2018 [265]
Kidney (2 mm-thick tissue slices)	0.02% Trypsin + 2% Tween-20 + 4% Sodium deoxycholate + 1% SDS	Seeding of renal stem/progenitor-like cells (nephrosphere)	-	Bombelli et al., 2018 [261]

SDS, sodium dodecyl sulfate; DNase, deoxyribonuclease; + means that separate cycles were performed; ‘and’ means that a mixture was performed between different substances.

**Table 10:** Reproductive tissues. Decellularization techniques, biomechanical tests, recellularization methods, and in vivo implant of human urogenital extracellular matrices.

Tissues	Decellularization Methods	Biomechanical Tests	In Vitro Recellularization	In Vivo Implant	References
Testis	1% Triton X-100	-	-	-	Baert et al., 2015 [272] Baert et al., 2017 [273]
	1% SDS (*)		Seeding of human testicular cells from orchidectomy		
	1% Triton X-100 + 1% SDS		-		
Glans	1% Triton X-100 and 0.1% ammonium hydroxide	Uniaxial tensile test	Perfusion of rat mesenchymal stem cells	-	Egydio et al., 2015 [275]
Corpus cavernosum	1% Triton X-100 + 5% SDS	-		Rats: sections of decellularized scaffold transplanted into the omentum and located into the scrotum	Kajbafzadeh et al., 2017 [276]
Urethra and Corpus spongiosum	1% Triton X-100 + 3% SDS	Uniaxial tensile test	Seeding or perfusion of preputial mesenchymal stem cells	In vivo implant according to Kajbafzadeh et al., 2017 [276]	Kajbafzadeh et al., 2017 [277]
Penile tunica albuginea	PEG 1000	Uniaxial strength test	-	-	da Silva et al., 2011 [274]
	Triton X-100				
Prostate	5 mM EDTA and 10% dimethyl sulfoxide + 1% TrytonX-100 + 0.5 M NaCl + 10 mM sodium cholate + 50 mM Tris-HCl + 100 U/mL Benzonase^®^	-	Seeding of primary prostate cancer cells		Cazzaniga et al., 2016 [278]
Ovary	1% sodium lauryl ester sulfate + DNase I	-	Seeding of rat primary ovarian cells	Immature female rats: acellular and recellularized scaffolds onto the renal fat pad after ovariectomy	Hassanpour et al., 2018 [279]
Myometrium	70% Ethanol + 0.25% Trypsin/EDTA	-	Seeding of human myometrial cells	-	Young and Goloman, 2013 [280]
Endometrium	0.25% Triton X-100 and 0.25% SDS + ribonuclease and DNase I	-	Seeding of human endometrial cells	-	Olalekan et al., 2017 [281]

PEG, polyethylene glycol; SDS, sodium dodecyl sulfate; + means that separate cycles were performed; ‘and’ means that a mixture was performed between different substances; (*) best protocol.

**Table 11:** Wharton’s jelly. Decellularization techniques, biomechanical tests, recellularization methods, and in vivo implant of human Wharton’s jelly extracellular matrix.

Tissue	Decellularization Methods	Biomechanical tests	In Vitro Recellularization	In Vivo implant	References
Wharton’s jelly	dH_2_O + 4% sodium deoxycholate + 2000 KU DNase-I + homogenation in 10% acetic acid solution (2.5 M)	-	Seeding of human primary chondrocytes	-	Stocco et al., 2014 [36]
	Hypertonic salt solution + hypotonic solution (0.005% Triton X-100) + anionic detergent (sodium lauryl) and sodium succinate + Benzonase^®^ + 40% ethyl alcohol	-	Seeding of: - human Wharton’s jelly mesenchymal stem cells; - bone marrow mesenchymal stem cells	Full-thickness parietal bone defect—craniotomy on transgenic mice.	Jadalannagari et al., 2017 [283]
	TBS 10 mM and 0.1% *w*/*v* EDTA + 0.03% SDS in TBS and EDTA	Compressive and tensile properties	Seeding of human fibroblasts cell line (HSF-PI 18)	Regeneration of full-thickness wound in mice	Beiki et al., 2017 [284]
	0.05% Triton X-100 + hypertonic salt solution + 250 U/µL Benzonase^®^ + N-lauroylsarcosine + ethanol solution + saline mannitol solution	-	Seeding of: - purified umbilical cord blood hematopoietic stem and progenitor cells; - leukemia cell lines: HL60, Kasumi I and MV 411; - primary bone marrow stromal cells	-	Converse et al., 2017 [287]

dH_2_O, deionized water; TBS, Tris buffered saline; EDTA, ethylene-diamine-tetra-acetic acid; SDS, sodium dodecyl sulfate; + means that separate cycles were performed; ‘and’ means that a mixture was performed between different substances.

**Table 12:** Placenta. Decellularization techniques, biomechanical tests, recellularization methods, and in vivo implant of human placenta extracellular matrix.

Tissue	Decellularization Methods	Biomechanical Tests	In Vitro Recellularization	In Vivo Implant	References
Placenta (Intact and highly vascularized portion)	Cyclic perfusion or soaking with these solutions: *(A) hypotonic Tris solution:* 10 mM Tris base, 5 mM EDTA, 1% PMSF + *(B) detergent extraction:* 50 mM Tris base, 1.5 M KCl, 5 mM EDTA, 1% lauroyl sarcosine, 1% PMSF *(C) detergent extraction:* 50 mM Tris base, 1% lauroyl sarcosine Two enzymatic digestion phases in 15,000 U DNAse Type II, 12.5 mg RNAse Type III A also occurred.	-	Seeding of primary human adipose precursor cells	-	Flynn et al., 2006 [287]
Placenta (Intact and highly vascularized portion)	Perfusive and diffusive protocol according to Flynn et al., 2006 [209]	-	Seeding of primary human adipose precursor cells	-	Flynn et al., 2007 [288]
Placenta (entire)	Homogenation in dH_2_O + 0.5% SDS + dH_2_O + 0.2% DNase (2000 U) and 200 μg/ml RNase	Tensile testing	-	Cutaneous wounds in rats	Choi et al., 2013 [289]
Placenta (entire)	Perfusion with: SDS 0.01% + 0.1% + 1% + dH_2_O + 1% Triton X-100	-	-	-	Kakabadze and Kakabadze, 2015 [290]
Placental vessels	FT + Perfusion with: 1.2% NaCl (hypertonic solution) + 0.4% NaCl (hypotonic solution) + 1% Triton X-100 + 0.02% *w*/*w* EDTA + DNAse I (200 IU/mL)	Tensile testing	Seeding of human umbilical vein endothelial cells	-	Schneider et al., 2016 [9]
Placenta (entire)	2% lauryl sarcosine + homogenation in 0.1 HCl with pepsin	-	Seeding of: - cardiomyocytes differentiated from induced pluripotent stem cells; - adipose stem cells, - human umbilical vein endothelial cells	Rat acute myocardial infarction model	Francis et al., 2017 [292]
Placenta (entire)	Perfusion with: 0.01% + 0.1% + 1% SDS + dH_2_O + 1% Triton X-100	Evaluation of mechanical strength of vessels	-	Heterotopic transplantation of hepatized placenta in sheep	Kakabadze et al., 2016 [291]
Placental vessels	Perfusion with:	-	Seeding of macrophages	Implantation of grafts into the infrarenal aorta of rats	Schneider et al., 2018 [9]
	1% Triton X-100 and 0.02% *w*/*w* EDTA + heparin cross-linking				
	0.5% SDS and 0.02% *w*/*w* EDTA + heparin cross-linking				

EDTA, ethylene-diamine-tetra-acetic acid; PMSF, phenylmethylsulfonyl fluoride; dH_2_O deionized water; SDS, sodium dodecyl sulfate; + means that separate cycles were performed; ‘and’ means that a mixture was performed between different substances.

**Table 13:** Cornea. Decellularization techniques, biomechanical tests, recellularization methods, and in vivo implant of human corneal extracellular matrix.

Tissues	Decellularization Methods	Biomechanical Tests	In Vitro Recellularization	In Vivo Implant	References
Corneal stroma (120–200 µm thickness slices)	2% Triton X-100 and 0.1% NH_4_OH	Uniaxial tensile tests	Seeding of human corneal endothelial cells	-	Choi et al., 2010 [322]
Cornea	0.1–1% Triton X-100	-	Seeding of: - human corneal epithelial cells - fibroblasts	-	Shafiq et al., 2012 [327]
	0.1–1% SDS				
	liquid nitrogen + hypoxic environment				
	PEG				
	10.0mM Tris + 1% Triton X-100, 1.5 M KCl, and 10.0 mM Tris + 1% SDS and 10.0 mM Tris + 1% Triton X-100				
	1.5 M NaCl + 5 U/mL DNAse and 5 U/mL RNAse (*)				
Corneal stroma (90 µm thick sheets)	1% SDS and protease inhibitors + 6.5 U/mL DNAse	-	Seeding of human adipose derived adult stem cells	Rabbits: recellularized corneal sheets into the cornea	Alio del Barrio et al., 2015 [329] Alió del Barrio et al., 2018 [79]
Cornea	2% Triton X-100 and 0.1% NH_4_OH +/− 5 U/mL DNase	-	Seeding of: - human corneal endothelial cells - human limbal epithelial cells	-	Zhang et al., 2015 [326]
	1.5 M NaCl +/− 5 U/mL Dnase				
Corneo-scleral rims	100mM EDTA + mechanical abrasion of the epithelium	-	Transplantation of human limbal epithelial cell sheets onto the limbus of decellularised corneal scleral rims	-	Genicio et al., 2015 [328]
Cornea	1.5M NaCl +/− 5 U/mL DNase and 5 U/mL Rnase	-	-	-	Wilson et al., 2016 [324]
	0.5% SDS +/− 5 U/mL DNase and 5 U/mL RNase				
	1% *w*/*v* Triton-X100 +/− 5 U/mL DNase and 5 U/mL RNase				
	2.4 U/mL Dispase II +/− 5 U/mL DNase and 5 U/mL Rnase				
	Mechanical agitation +/− 5 U/mL DNase and 5 U/mL RNase				
Corneal stromal lenticules	1.5 M NaCl + Mechanical agitation	-	Seeding of primary human stromal fibroblasts	Rabbits: decellularized 70 μm lenticules into a corneal stromal pocket	Yam et al., 2016 [331]
	0.1% SDS (*)				
	0.1% Triton X-100				
	0.1% SDS and 0.1% Triton X-100				
	1.5 M NaCl + 2; 5; 10 U/mL nuclease				
	1.5 M NaCl + 2; 5; 10 U/mL nuclease + 0.1% SDS				
Corneal lamellae	Mechanical agitation (sonification)	-	Seeding of human corneal endothelial cell line	-	He et al., 2016 [330]
	freezing/thawing (liquid nitrogen/37 °C)				
	freezing in liquid nitrogen + hypoxia in nitrogen				
	1.5 M NaCl + 0.02% EDTA/0.05% trypsin				
	0.1% SDS				
	1% SDS + Mechanical agitation				
	1% SDS + DNase + Mechanical agitation (*)				
Corneal stromal lenticules	1.5 M NaCl + 5 U/mL DNAse and 5 U/mL RNAse	-	-	Rabbits: fibrin glue-adhered lenticules into the corneal stroma	Yin et al., 2016 [332]
Cornea	freezing/thawing	-	-	Rabbits: Descemet’s membrane transplantation into the wounded corneal endothelium	Bhogal et al., 2017 [333]

SDS, sodium dodecyl sulfate; PEG, poly(ethylene glycol); DNase, deoxyribonuclease; RNase, ribonuclease; + means that separate cycles were performed; ‘and’ means that a mixture was performed between different substances: +/− means that the treatment was repeated with or without the nuclease incubation; (*) best protocol.
